# Genes in the terminal regions of orthopoxvirus genomes experience adaptive molecular evolution

**DOI:** 10.1186/1471-2164-12-261

**Published:** 2011-05-23

**Authors:** David J Esteban, Anne P Hutchinson

**Affiliations:** 1 Biology Department, Vassar College, 124 Raymond Ave, Poughkeepsie, NY, 12604, USA

## Abstract

**Background:**

Orthopoxviruses are dsDNA viruses with large genomes, some encoding over 200 genes. Genes essential for viral replication are located in the center of the linear genome and genes encoding host response modifiers and other host interacting proteins are located in the terminal regions. The central portion of the genome is highly conserved, both in gene content and sequence, while the terminal regions are more diverse. In this study, we investigated the role of adaptive molecular evolution in poxvirus genes and the selective pressures that act on the different regions of the genome. The relative fixation rates of synonymous and non-synonymous mutations (the d_N_/d_S _ratio) are an indicator of the mechanism of evolution of sequences, and can be used to identify purifying, neutral, or diversifying selection acting on a gene. Like highly conserved residues, amino acids under diversifying selection may be functionally important. Many genes experiencing diversifying selection are involved in host-pathogen interactions, such as antigen-antibody interactions, or the "host-pathogen arms race."

**Results:**

We analyzed 175 gene families from orthopoxviruses for evidence of diversifying selection. 79 genes were identified as experiencing diversifying selection, 25 with high confidence. Many of these genes are located in the terminal regions of the genome and function to modify the host response to infection or are virion-associated, indicating a greater role for diversifying selection in host-interacting genes. Of the 79 genes, 20 are of unknown function, and implicating diversifying selection as an important mechanism in their evolution may help characterize their function or identify important functional residues.

**Conclusions:**

We conclude that diversifying selection is an important mechanism of orthopoxvirus evolution. Diversifying selection in poxviruses may be the result of interaction with host defense mechanisms.

## Background

Poxviruses are a family of double stranded DNA viruses that infect diverse host species. The genus *Orthopoxvirus *includes Variola (the causative agent of smallpox), Vaccinia (the smallpox vaccine), Monkeypox, an emerging human pathogen, and Cowpox.

Poxviruses are among the largest and most complex of all animal viruses, some expressing over 200 genes [1]. A large fraction of the coding capacity of the genome is for processes essential for viral replication, such as virion assembly, transcription and replication. Unlike other DNA viruses, poxviruses replicate in the cytoplasm and therefore encode all genes necessary for DNA replication and transcription. These essential genes are highly conserved throughout the poxvirus family and in orthopoxviruses these core genes form a continuous block in the center of the linear genome [2,3].

Flanking the central region, the terminal regions of orthopoxvirus genomes show divergence among different genera, among species within a genus, and even among strains of the same species [4]. Many of these genes are non-essential for virus replication in cell culture but are virulence factors that mediate interactions with the host cell or immune system in their natural host. These include immune evasion genes that inhibit cytokines [5], inhibit the interferon response [6], or block apoptosis [7]. Many of these are host species specific, indicating adaptation to the specific host response to infection.

Analysis of the content and organization of the orthopoxvirus genome implicates gene gain and loss as major mechanisms in their evolution [8]. Cowpox virus strain Brighton Red (CPXV-BR) has a "master set" of genes; all other orthopoxviruses have a smaller subset of those genes. Thus it is likely that as the orthopoxviruses evolved and diversified into different hosts, some genes were lost while those that were retained adapted to the specific host. The sequence conservation of the core genes may be the result of stringent structural or functional constraints on these core proteins. Host-response modifier genes in the terminal regions, however, may be more able to change and thus show greater sequence divergence. As such, we were interested in understanding the role of adaptive molecular evolution in poxvirus genes and the selective pressures that act on genes in different regions of the genome. Adaptive molecular evolution, or diversifying selection, is a key mechanism for species divergence and identifying proteins or specific residues experiencing diversifying selection may be important in understanding gene function.

Diversifying selection can be detected by measuring the ratio of non-synonymous/synonymous mutation fixation rates (ω = d_N_/d_S_) [9-11]. Codons experience continual nucleotide substitutions as a result of errors in replication. Substitutions that result in synonymous mutations are selectively neutral. However, non-synonymous mutations can occur and become fixed as a result of selection. In purifying selection (ω < 1), a higher rate of synonymous than non-synonymous mutations occurs, suggesting stringent structural or functional constraints on the amino acid. In neutral selection (ω = 1) a change to a different amino acid has neither a positive nor negative effect on the protein. In diversifying selection (ω > 1) the rate of fixation of non-synonymous mutations is greater than the rate of fixation of synonymous mutations, indicating adaptive molecular evolution.

In this study we analyzed a set of 175 orthopoxvirus gene families using CPXV-BR as a reference genome to determine the selective pressures acting upon them. We show that diversifying selection occurs most strongly in the terminal regions of the genome. The results of this study may help identify important functional regions in genes of known or unknown function, and may help categorize genes of unknown function as host-interacting genes.

## Results

### Gene families

The data set collected from the Viral Bioinformatics Resource Centre database contained 19,874 chordopoxvirus gene sequences. After the removal of identical sequences, 7758 unique sequences from 103 viruses remained (Additional file 1). Sequences were parsed into 380 gene families of which 214 were present in the CPXV-BR genome.

The CPXV-BR genome encodes 235 genes belonging to 214 gene families. Of the 214 gene families, 38 were excluded from the analysis (Additional file 2). 16 gene families (containing a total of 37 genes) were excluded because they were duplicated in the genome and therefore have the potential for differential evolutionary pressures on each paralog. 17 families with fewer than 6 sequences were excluded, since too few taxa in the dataset results in poor power to identify amino acid sites under diversifying selection [12]. Gene family size in the final dataset ranged from 6-69 sequences. 6 gene families were removed because of difficulty in generating a reliable alignment, fragmentation of the gene in CPXV, or excessive computational time required for analysis due to large family size and length of genes. The final dataset had a total of 175 gene families, comprising a total of 6058 sequences analyzed (Additional file 3). CPXV-BR is used as the reference strain for the purposes of referring to gene names, codon positions and genome organization, however, because the analysis is performed on gene families, not single gene sequences, the results apply to homologous genes of all poxviruses used in this study.

Treelength (S), measured as the number of substitutions per codon in the tree, can be used as a measure of sequence divergence in the gene family. Genes with treelengths that are too short (S < 0.11) are insufficiently divergent for this analysis [12]. All genes included in this analysis had S > 0.11. S values ranged from 0.225 to 43.02, with only three above 30 (Additional file 3). Genes found to be under diversifying selection (see below) were distributed throughout the range of treelengths.

The length of CPXV genes ranged from 42 to 1286 codons, with the majority having a length of less than 400 codons, and only 5 greater than 800 codons. Genes found to be under diversifying selection were distributed throughout the range (data not shown).

### Diversifying selection

We wanted to determine which genes in poxvirus genomes showed adaptive molecular evolution (diversifying selection). To do this, we used site models of codon evolution to determine the rate of fixation of synonymous and non-synonymous mutations that can identify the presence of an amino acid site (codon) class under diversifying selection. Codon models use a statistical distribution to describe the variation in ω among sites, and make no assumptions about which sites are under diversifying selection. Site models permit differing d_N_/d_S _ratios at each codon, and because selective pressures are expected to vary across amino acids, this approach has high power to detect diversifying selection in genes [11]. Each gene family was analyzed for evidence of diversifying selection by testing if the data fits a null model (which does not allow for sites under diversifying selection) better than an alternative model, which does allow for sites under diversifying selection. Two model comparisons were applied: M1a vs. M2a, M7 vs. M8, where M1a and M7 are null models and M2a and M8 are alternate models. A log likelihood ratio test was then applied to determine whether allowing for a site class with ω>1 results in a significantly better fit of the model to the data.

Evidence for diversifying selection was considered to be strong where both comparisons were found to be significant at p < 0.05. 25 genes were identified by both models as having an amino acid site class experiencing diversifying selection (null models rejected, p < 0.05) (Table 1). With model M8 (a less conservative model) an additional 54 genes were identified as having sites experiencing diversifying selection (null model rejected, p < 0.05), for a total of 79 genes under diversifying selection (Additional file 4). All significant genes under model M2a were also significant under model M8, except one (Unknown YMTV120.5L).

**Table 1:** Genes under diversifying selection identified by both models

ORF Number	Gene Family Name	Category	VACV-Cop ORF	Specific Function	Virion
021	EGF_Growth factor	Host Response Modifier	C11R	EGF homolog with mitogenic activity	No

024	IL_18_BP (Bsh_D7L)	Host Response Modifier	n/a	Inhibition of IL-18 activity	No

035	Kelch_like_ (Cop_C2L)	Host Response Modifier	C2L	Unknown	No

036	Unknown (Cop_C1L)	Unknown	C1L	Unknown	No

046	Unknown (Cop_K7R)	Entry/Exit	K7R	inhibitor of pattern recognition receptor signalling pathway	No

048	Apoptosis inhibitor (mitochondrial associated)	Host Response Modifier	F1L	localizes to mitochondria and inhibits apoptosis	No

054	Unknown (Cop_F6L)	Unknown	F6L	Unknown	No

055	Unknown (Cop_F7L)	Unknown	F7L	Unknown	No

056	Cytoplasmic protein (Cop_F8L)	Unknown	F8L	Cytoplasmic localization, unknown function	No

063	Unknown (Cop_F14L)	Unknown	F14L	Unknown	No

129	Carbonic anhydrase Virion	Entry/Exit	D8L	Binds Chondroitin Sulfate on cell surface	Yes

140	Core_protein (Cop_A4L)	Structure/Assembly	A4L	associated with virion core and membranes	Yes

145	Membrane protein (Cop_A9L)	Structure/Assembly	A9L	located on virion membrane, essential for morphogenesis	Yes

162	RNA pol 132 (RPO132)	RNA metabolism	A24R	Component of RNA polymerase	Yes

171	Unknown (YMTV_120.5L)	Unknown	A30.5L	Unknown	No

172	Unknown (Cop_A31R)	Host Response Modifier	A31R	Unknown	Yes

182	Semaphorin	Host Response Modifier	A39R	Induces IL-6 and IL-8 secretion from monocytes	No

191	Unknown (Cop_A47L)	Unknown	A47L	Unknown	No

192	Thymidylate kinase	DNA metabolism	A48R	Thymidylate kinase activity	No

200	Hemagglutinin	Host Response Modifier	A56R	prevents cell-cell fusion, present on EEV and cell surface	Yes

203	Schlafen (Cop_B2R)	Host Response Modifier	B2R	Virulence factor in CMLV	No

204	Ankyrin (Cop_B4R)	Unknown	B4R	Unknown	No

212	Ser_Thr_Kinase (Cop_B12R)	Unknown	B12R	Unknown	No

215	IL_1_beta receptor	Host Response Modifier	B15R	Inhibits IL-1 beta activity	No

216	Unknown (Cop_B17L)	Unknown	B17R	Unknown	No

Alternative models M2a and M8 allow for the existence of a codon site class under diversifying selection. The proportion of sites that fall into this site class provides insight into the selective pressure experienced by the gene. For each gene, the proportion of sites that are under diversifying selection was determined (Additional file 5). Among the genes for which the diversifying selection models showed a significantly better fit of the data, the proportion of sites under diversifying selection ranged from 0.1% to 14.6% (model M2a) and 0.1% to 29.5% (model M8). A gene does not necessarily need a large proportion of sites under diversifying selection to fit the alternative model better. In many genes, a large proportion of amino acids may be virtually invariable due to structural or functional constraints, while diversifying selection occurs at only a few key sites. For example, the F6L homolog (unknown function) has a small proportion of sites under diversifying selection (2.9%), while the mitochondrial associated apoptosis inhibitor has a large proportion of sites under diversifying selection (14.0%) and for both the null model was rejected.

**Figure 1:**
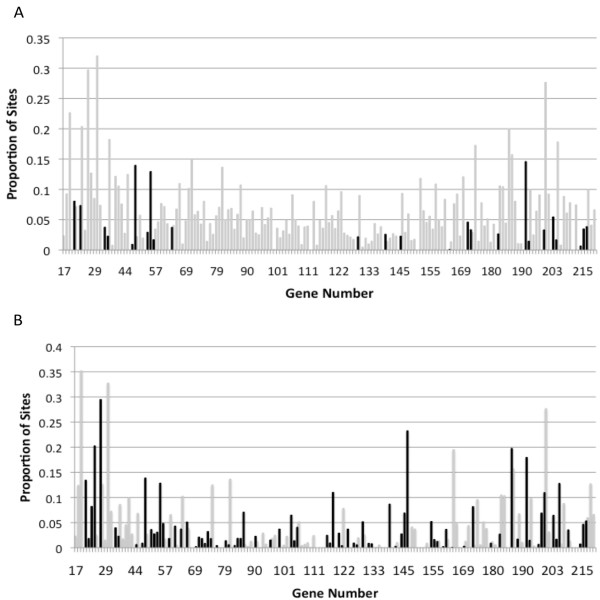
**Proportion of sites under diversifying selection in each gene in the CPXV genome **. Black: Genes with sites under diversifying selection (Genes for which the alternative model, allowing positively sites with ω > 1, fits the data better than the null model (p < 0.05)). White: Genes without sites under diversifying selection. A) Proportions determined using model M2a and LRT against M1a. B) Proportions determined using model M8 and LRT against M7.

Using the CPXV-BR genome as a reference for gene positions within the genome, we noted a distinct pattern of genes with a high proportion of sites under diversifying selection being located closer to the termini of the genome, as well as a tendency for significant genes (model M2a) to be located in the terminal regions (Figure 1). A centrally located core set of 90 genes is present in all chordopoxvirues [2]. A chi square test revealed that genes experiencing diversifying selection identified by model M2a were significantly more likely to be found among the 85 terminally located less conserved genes (χ^2 ^= 12.56, df = 1, p < 0.05). The 79 genes identified by model M8 however were proportionately distributed between the core and less conserved genes (χ^2 ^= 0.05, df = 1, p > 0.05).

**Figure 2:**
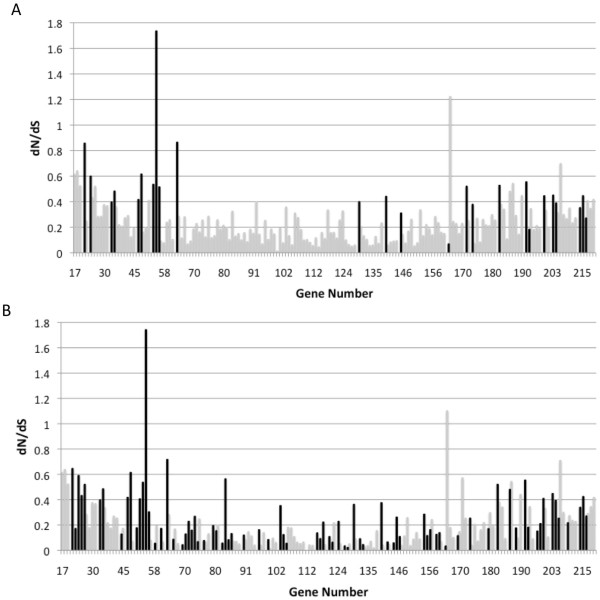
**d**_**N**_**/d**_**S **_**(ω) ratios of genes in the CPXV genome **. The ratio for the gene is the average ω of all codons in the gene. Black: Genes with sites under diversifying selection (Genes for which the alternative model, allowing positively sites with ω>1, fits the data better than the null model (p < 0.05)). White: Genes without sites under diversifying selection. A) Ratios determined using model M2a, with statistical significance determined by comparison with M1a. B) Ratios determined using model M8, with statistical significance determined by comparison with M7.

The non-synonymous/synonymous rate ratio (ω = d_N_/d_S _) is an important indicator of selective pressure at the protein level. The d_N_/d_S _ratio of a gene is calculated as the average of the ω of each codon in the gene. A higher d_N_/d_S _ratio suggests a greater role for diversifying selection in the molecular evolution of the gene. Again we note that genes with high d_N_/d_S _ratios are located in the termini of the genome (Figure 2). Some significant genes have an average d_N_/d_S _ratio less than 1, indicating that diversifying selection has occurred in these genes, but not sufficiently to cause the average to be greater than 1. These genes may have only a small proportion of amino acids under diversifying selection or a larger proportion that experiences weaker diversifying selection.

The d_N_/d_S _ratio of the site class under diversifying selection (ω2) was also determined (Additional file 5). This value is a measurement of the intensity of diversifying selection within that site class. For example, Semaphorin (CPXV-BR-182) experiences very strong diversifying selection (ω2 = 10.417) in only a small proportion of total sites (2.7%). The F7L homolog (CPXV-BR-055, unknown function) also has a site class experiencing diversifying selection (ω2 = 8.343), however this slightly weaker selection is spread over a greater proportion of sites (12.9%). For this reason, diversifying selection cannot be identified solely on the basis of quantity of sites experiencing diversifying pressure along the gene, but also the strength of the pressure acting on these genes. A few genes have ω2 > 1 but the data do not fit the diversifying selection model significantly better than the null model, indicating that a site class under diversifying selection likely does not exist for these genes.

### Function and localization of genes under diversifying selection

**Figure 3:**
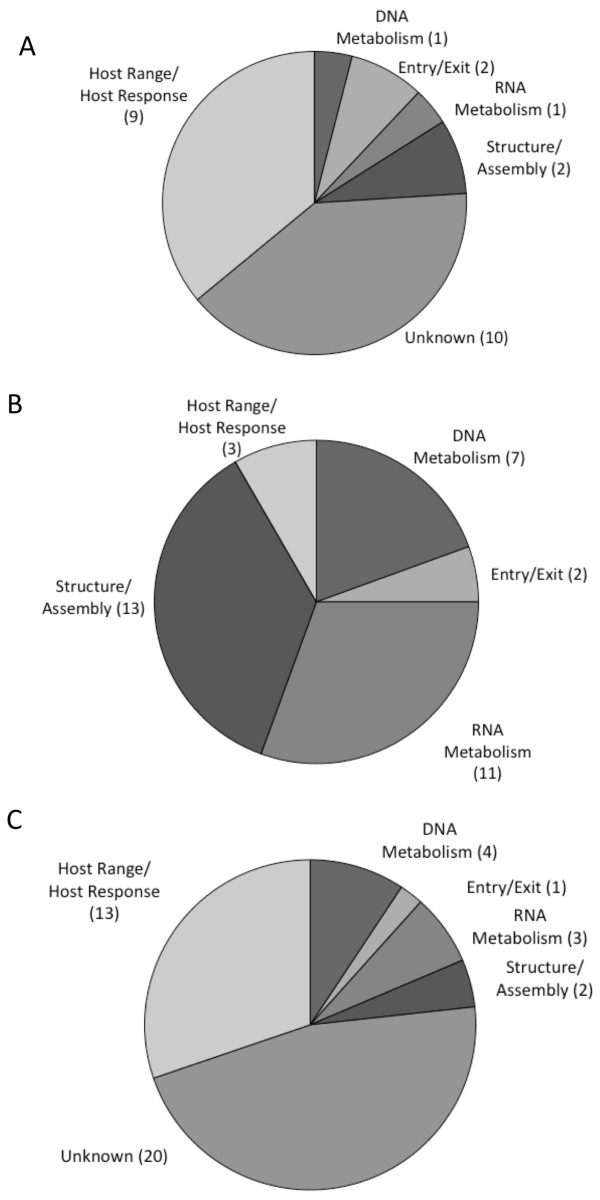
**Functional categories of the genes under diversifying selection **. A) Functions of the 25 genes identified by both models. B) Functions of 43 non-virion-associated proteins identified by model M8, B) Functions of 36 virion-associated proteins identified by model M8.

The 79 genes identified were broadly categorized by function and localization (Table 1, Additional file 4 and Figure 3) based on annotation in the Viral Bioinformatics Resource Center curated genes database [13] and several reports on poxvirus genomes [1,14,15]. The largest category is that of genes of unknown function (20 genes), but many host response modifiers or host-range genes (16 genes) were also found. Enzymes and structural components were also identified. Classified by localization, 36 of the proteins under diversifying selection have been found to be virion associated (Figure 3B and 3C). Localization in the virion was determined from two proteomics studies [16,17] and previous literature [1]. All of the proteins of unknown function, and most (81%) of the host response modifiers are not found in the virion. 18 out of 25 (72%) of the enzymes involved in DNA or RNA metabolism, as well as the majority of proteins used to assemble or form the structure of the virion (87%) are found in the virion. Focusing on the 25 genes identified by both models, host-response modifiers and genes of unknown function make up the largest proportions, 36% and 40%, respectively (Figure 3A). Of the 6 remaining genes, 5 are found in the virion (Thymidine kinase is not found in the virion).

### Identification of specific residues under diversifying selection

**Figure 4:**
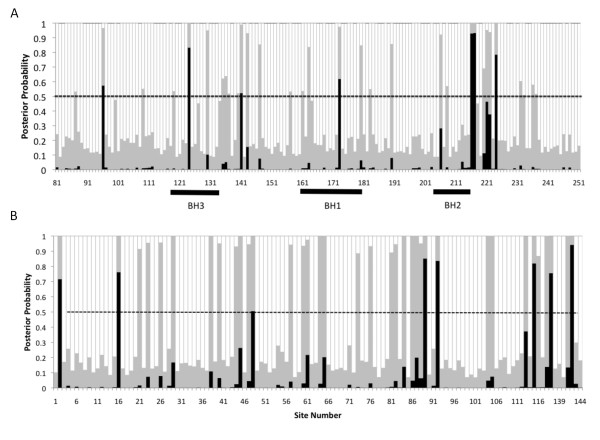
**Posterior probability of site classes for amino acids in two host response modifier genes **. Shown for each site is the posterior probability of falling into the purifying (white), neutral (grey), or diversifying (black) class. Posterior probabilities were determined by Bayes empirical Bayes analysis. Dashed line at 0.5 shows the threshold for a site to be classified as diversifying. A) IL-18 binding protein. B) mitochondrial associated apoptosis inhibitor. Only the conserved C-terminal domain is shown, and the locations of the BH domains are indicated below the axis.

For each gene identified as experiencing diversifying selection, the posterior probabilities of each site falling into the three categories were calculated using a Bayes empirical Bayes (BEB) analysis [18]. For each gene, every codon was assigned to either the purifying, neutral or diversifying site classes. Two examples are shown: the Interleukin-18 binding protein (IL-18BP) (Figure 4A) and the mitochondrial associated apoptosis inhibitor (Figure 4B). As with most genes, most codons of the IL-18BP gene are under purifying selection, with a small proportion under neutral or diversifying selection. For example, the posterior probabilities for site W124 are 0.000, 0.057, and 0.942 for purifying, neutral and diversifying selection, thus the site is highly likely to be under diversifying selection. A total of 8 specific sites are predicted to be under diversifying selection in IL-18BP, and 7 sites are under diversifying selection in the highly conserved C-terminal domain of the mitochondrial associated apoptosis inhibitor, and an additional 2 are in the N-terminal region. For other genes, the specific residues with a high posterior probability of falling into the diversifying selection site class are given in Additional file 6 and Additional file 7.

## Discussion

In this study we analyzed a large set of poxvirus genes with the purpose of identifying genes experiencing adaptive molecular evolution. Of 235 genes in the Cowpox virus genome, 175 were analyzed using phylogenetic analysis of maximum likelihood (PAML) and subsequently analyzed by Bayes empirical Bayes to determine the probability of each codon falling into the three possible site classes of purifying, neutral or diversifying selection. We identified 79 genes under diversifying selection, representing 45% of the analyzed genes in the genome. Of those, 25 (14% of genes analyzed) were identified with both models, indicating high confidence. Thus we identify diversifying selection as an important mechanism of evolution in poxviruses.

Analysis of genes spanning the CPXV genome revealed that diversifying selection is a more important mechanism of molecular evolution in the genome's terminal regions than in the central genes. This may be due to diversifying pressures applied by host interactions. Among different orthopoxvirus species and even strains, terminal regions are more diverse both in gene content and gene sequences [2]. Many terminally located genes are host response modifiers that directly interact with components of the host immune response or cellular response to infection. Grouping the genes identified in this study into broad functional categories (Figure 3), we identified several host response modifiers and host range genes that have sites under diversifying selection. These may be sites that are involved in host-specific interactions, demonstrating adaptation to the virus's particular host. Previous studies have also demonstrated diversifying selection in poxvirus host response modifying genes [19-21]. The current study uses the largest dataset providing the most comprehensive analysis of diversifying selection in poxvirus genomes. Among the host response modifiers identified were several secreted immunomodulators (IL-18 binding protein, IL-1 beta receptor and IFN gamma receptor) that are known to contribute to virulence [22-24]. Genes that modulate the cellular response to infection were also identified, including the mitochondrial associated apoptosis inhibitor, an inhibitor of the protein kinase R (PKR) response to double stranded RNA, a ubiquitin ligase, and an inhibitor of Toll-like receptor signaling [25-28].

In addition to host response modifying genes, we also showed, using model M8, that genes involved in viral replication and virion structure experience diversifying selection, possibly due to their protein products being packaged in the virion. A proteomics study [17] identified 75 viral proteins in the VACV virion, 18 of which are present at abundances greater than 1% of the weight of the virion. Of those, 6 were identified in the current study, including the major core protein (A4), the most abundant protein in the virion by weight. Further, the immune response to poxvirus infection induces the formation of neutralizing antibodies to several virion membrane proteins [29,30]. We identified 3 known major targets of neutralizing antibodies: an immature virion membrane protein (A7, CPXV-BR-154), carbonic anhydrase (D8, CPXV-BR-129) and an enveloped virion protein (B5, CPXV-BR-205). Thus detection of diversifying selection is not limited to host response modifying genes but more broadly to genes whose products interact with the host, such as major antigens.

Designation of families in the VOCs database was based on a BLAST expect value of 10^-17^, thus families are likely to be composed of orthologs [2]. Horizontal gene transfer (HGT) is recognized as a factor in evolution of poxvirus genomes. It is possible that some of the genes within a gene family may not be orthologs if they arose through multiple independent horizontal gene transfer events. Several lines of evidence are needed to demonstrate HGT, including phylogenetic clustering of the gene in taxa unrelated to the genome under study. The origins of most poxvirus genes are unknown, although most chordopoxvirus genes show greater similarity to eukaryotic genes than to other viral genes [31]. Full phylogenies of each poxvirus gene family are needed to determine if multiple horizontal gene transfer events have occurred within the family. Such research is underway and will be valuable in further interpretation of the current data.

Like highly conserved amino acids, variable positions under diversifying selection may indicate functionally or structurally important positions. Computational identification of amino acids under diversifying selection has revealed important functional or antigenic sites in several studies [32,33]. One of the genes identified in this study was the Interleukin-18 binding protein (IL-18BP), which was previously shown to attenuate the immune response in mice [22]. The crystal structure of the Ectromelia virus (ECTV) IL-18 binding protein was recently determined [34]. Bayes empirical Bayes analysis demonstrated that most residues are under purifying selection, while some are neutral and a few are under diversifying selection (Figure 4). The crystal structure and previous mutagenesis studies [35] identified contact residues important in binding the ligand, IL-18. Most of these residues were found to be under purifying selection, supporting a requirement for conservation of these residues to maintain function. Interestingly, 2 residues shown through crystallography to be contacts in the binding interface were also identified as being under diversifying selection (D48 and I115 in CPXV-BR, E48 and L115 in ECTV). Both of these residues interact with binding site C on human IL-18 [34]. Evidence of diversifying selection in these positions may suggest a role for these residues in adaptation to IL-18 of the specific host species of the virus.

Another important host response modifying gene that we identified is the mitochondrial associated apoptosis inhibitor (F1L in VACV-Cop). Apoptosis is an important cellular response to infection that serves to limit viral replication through removal of infected cells. F1L inhibits apoptosis through binding to the pro-apoptotic protein Bak [36,37] thereby inhibiting the permeabilization of the mitochondrial membrane, a critical step in apoptosis. Interaction with Bak is via Bcl2-like homology (BH) domains that are highly divergent but nonetheless form characteristic BH domain folds [37]. Among the poxviruses, the C-terminus (containing the BH domains) of the F1L family is highly conserved. There are 7 residues under diversifying selection located in this domain (Figure 4B). Of those, 2 residues identified in this study are located in the BH domains. BH3 and BH1, primarily located on alpha helices α2 and α5, make up the BH3 binding pocket responsible for binding Bak [37]. One residue (A173 CPXV) identified in this study, corresponding to A144 in the F1L homolog of Modified Vaccinia Ankara (MVA), is located in the binding pocket and was shown by mutagenesis to increase binding affinity if mutated to phenylalanine. M124 in CPXV (I95 in MVA) is located in α2 and therefore could be involved in ligand binding or in determining the shape of the pocket. Another 3 sites under diversifying selection are located immediately C-terminal to BH2. Overall, the capacity of the Bayes empirical Bayes analysis to identify residues known to be important in protein function, such as in the IL-18BP and mitochondrial associated apoptosis inhibitor, suggests that it may be valuable to test other predicted sites across the genome for their role in protein function.

The identification of host-interacting genes in poxviruses as ones experiencing adaptive molecular evolution is consistent with the findings of several other studies identifying genes involved in the "host-pathogen arms race" or other co-evolutionary processes and is seen throughout nature. Chitinase and other plant defense proteins show evidence of diversifying selection, and mutagenesis studies have confirmed the functional importance of the identified sites [32,38]. The *wsp *gene of the bacterium *Wolbachia*, which encodes an outer membrane protein, shows evidence of diversifying selection when in a parasitic relationship with arthropods, but not in a mutualistic relationship with nematodes [39]. In other viruses, all the major genes of HIV [40,41], and the capsid protein of Foot-and-Mouth Disease virus (FMDV) [33] experience diversifying selection. Importantly, the amino acids identified computationally in the FMDV capsid are known to be antigenic sites identified by monoclonal antibody escape mutants [33]. In host species, diversifying selection has been shown in the antigen recognition sites of the major histocompatibility (MHC) gene [42,43].

As has been demonstrated with the FMDV capsid protein [33], interaction between an antigen and the immune response can drive diversifying selection. Several studies have found evidence of diversifying selection in surface proteins on viruses, including HIV env, influenza hemagglutinin, and others [44-46]. Recent proteomics studies have identified the major and minor virion-associated proteins [16,17]. In this study, 39% of the genes identified are associated with the virion (Figure 3). Virion structural components and virion-associated enzymes may have greater exposure to antibody responses, leading to diversifying selection.

A few small whole genomes have been analyzed for diversifying selection, showing that diversifying selection can play strikingly different roles in the molecular evolution of organisms. A study of *Picornaviridae *shows evidence of diversifying selection in structural proteins but not in non-structural proteins [47]. Only a few codons in the astrovirus genome are under diversifying selection [48], while 9-38% of sites in human rhinoviruses experience diversifying selection [49]. The poxvirus genome is significantly larger than any other viral genome analyzed by this method, and the results indicate a more important role for diversifying selection in poxvirus genome evolution than in other viruses. This may be related to the large size of the poxvirus genome and the large number of accessory "non-essential" genes such as the host-response modifiers or the large number of encapsidated proteins. Over 1700 genes of the *Streptococcus *genome were analyzed and approximately 8% of the genes were found to be under diversifying selection [50]. Of those, 29% are related to virulence, and many others show tissue specific expression during invasive disease. A large fraction of poxvirus genes under diversifying selection are also known to be virulence factors, echoing the findings in *Streptococcus*.

In *Streptococcus*, several essential core function genes were identified, indicating that virulence is more complex than simply the presence of pathogen associated genes [50]. Similarly, analysis of *E. coli *genomes found 29 genes common to pathogenic and non-pathogenic strains which showed evidence of diversifying selection, and many of which are involved in functions like DNA metabolism and nutrient acquisition [51]. Several core poxvirus genes, not typically thought of as virulence factors, were also found to be under diversifying selection in the poxvirus genome. Some (but not all) of these are packaged in the virion and may be exposed to the host antibody response. However, this suggests that other poxvirus systems, in addition to manipulation of the host response, may be important in virulence. In attempting to explain virulence differences between strains of poxviruses, it may therefore be important to consider not only major genomic differences such as gene complement, but also diversifying selection in well conserved genes.

## Conclusions

The identification of 79 genes in the orthopoxviruses that experience diversifying selection implicates this as a major mechanism of poxvirus evolution. Because many of these genes either interact with host defense mechanisms or may be targets of the immune response, interaction with the host may be the basis for adaptive molecular evolution. Understanding the mechanisms of poxvirus evolution may shed light on important aspects of poxvirus biology such as adaptation of monkeypox virus to become more sustainable in human populations. Combined with experimental data, identification of sites under diversifying selection may be important in guiding future mutagenesis studies, as these residues may be important in host specific interactions such as protein-protein contacts or immune epitopes. Further, many genes with unknown or poorly defined functions were shown to have sites under diversifying selection. These data may provide a basis for investigating the function of these as host-interacting proteins or virulence factors, and may identify highly conserved functional residues or diverse host-specific residues that are of particular interest.

## Methods

### Sequence selection and alignment

A subset of viruses of the Chordopoxvirus subfamily was selected based on close phylogenetic relationships [4]. Fully sequenced genomes of the Orthopoxvirus, Leporipoxvirus, Yatapoxvirus, Capripoxvirus, Suipoxvirus and Mule Deer pox genera were used (Additional file 1, Table S1). Other Chordopoxvirus genera were excluded because their sequences were typically too divergent to generate reliable alignments and have very different %GC content compared to other poxviruses. Gene families were defined by the Viral Orthologous Clusters (VOCs) database [2]. CPXV-BR virus was used as a reference genome; all gene positions used are those in CPXV-BR, and only gene families with a member in CPXV-BR were used in the analysis. Gene families with duplicated genes (paralogs) and gene families that contained less than 6 genes were excluded.

All protein and nucleotide sequences were acquired from the VOCs database. Using perl scripts, identical nucleotide sequences and their corresponding amino acid sequences were removed and then parsed into their respective gene families. Codon based alignments of nucleic acid sequences of gene families were generated using PRANK [52]. PRANK has been shown to generate more reliable alignments resulting in fewer false positives than other alignment programs [53].

### Tree construction

Maximum likelihood trees were constructed from the nucleotide alignments using the DNAml executable within the PHYLIP program. The trees were used as starting points for subsequent analysis using PAML4.

### Detection of evolutionary pressures with PAML4

Each gene family was analyzed using the codeml program within the PAML4 package [54] to assess the selective forces acting on the genes. Codon based models were used since they have the greatest power to detect differing selective pressures acting on a protein [10,11]. To identify the presence of a codon site class under diversifying selection, two separate site model comparisons were used. In the first, the null model M1a, which allows for two site categories, ω = 0 and ω = 1, was compared to the alternative model M2a, that has an additional site class in which ω is allowed to exceed 1. In the second comparison, the null model (M7) that allows for sites with 0 < ω < 1, was compared to the alternative model (M8) that allows for an additional site class in which ω may exceed 1. Each gene family was analyzed on a 156 core cluster composed of 2.2GHz AMD Opteron cores. The computational time taken for each gene family varied by the number of sequences per gene family and the length of the sequences and ranged from several minutes to 31 days. Perl scripts were then used to compile results.

Differences in log likelihood values between null and alternative hypotheses were analyzed using the Likelihood Ratio Test (LRT) and the resulting values compared to appropriate chi squared values to determine significance, using a confidence threshold of p < 0.05 and df = 1.

### Bayes empirical Bayes

For genes in which the alternative model showed a significantly better fit of the data, Bayes theorem [18] was applied to calculate the posterior probability that each site belongs to a particular ω class. This was performed through the codeml executable of the PAML4 package [54].

## Authors' contributions

DJE conceived of the study, participated in its design and coordination, carried out the analysis and drafted the manuscript. APH participated in the design of the study, carried out the analysis, and helped to draft the manuscript. All authors have read and approved the final manuscript.

## Supplementary Material

Additional File 1 **Viral genomes used in this analysis**. Click here for file

Additional File 2 **Gene families excluded from analysis**. Click here for file

Additional File 3 **Basic statistics of genes used in analysis**. Click here for file

Additional File 4 **Genes under diversifying selection identified by model M8 only**. Click here for file

Additional File 5 **Proportions and omega values of significant genes**. Click here for file

Additional File 6 **Sites in significant genes (models M2a and M8) under diversifying selection determined by Bayes empirical Bayes analysis**. Click here for file

Additional File 7 **Sites in significant genes (model M8 only) under diversifying selection determined by Bayes Empirical Bayes analysis**. Click here for file
