# Supplementary material for: Genes in the terminal regions of orthopoxvirus genomes experience adaptive molecular evolution
Source: BMC Genomics. 2011 May 23;12:261. doi: 10.1186/1471-2164-12-261 (PMC3123329; doi:10.1186/1471-2164-12-261)
Supplement: Additional File 1 — Viral genomes used in this analysis. [file 1471-2164-12-261-S1.PDF]

Viral genomes used in this analysis

Genus	Species	Strain	Abbreviation	GenBank Accession
Capripoxvirus	Goatpox virus	G20-LKV	GTPV-G20LKV	AY077836
Capripoxvirus	Goatpox virus	Pellor	GTPV-Pellor	NC_004003
Capripoxvirus	Lumpy skin disease virus	Neethling 2490	LSDV-Nee	NC_003027
Capripoxvirus	Lumpy skin disease virus	Neethling vaccine LW 1959	LSDV-LW1959	AF409138
Capripoxvirus	Lumpy skin disease virus	Neethling Warmbaths LW	LSDV-NW_LW	AF409137
Capripoxvirus	Sheeppox virus	A	SPPV-A	AY077833
Capripoxvirus	Sheeppox virus	NISKHI	SPPV-NISKHI	AY077834
Capripoxvirus	Sheeppox virus	TU-V02127	SPPV-TU	NC_004002
Leporipoxvirus	Myxoma virus	6918	MYXV-6918	EU552530
Leporipoxvirus	Myxoma virus	Lausanne	MYXV-Lau	NC_001132
Leporipoxvirus	Rabbit fibroma virus	Kasza	RFV-Kas	NC_001266
Orthopoxvirus	Camelpox virus	CMS	CMLV-CMS	AY009089
Orthopoxvirus	Camelpox virus	M96	CMLV-M96	NC_003391
Orthopoxvirus	Cowpox virus	Brighton Red	CPXV-BR	NC_003663
Orthopoxvirus	Cowpox virus	Germany 91-3	CPXV-GER91	DQ437593
Orthopoxvirus	Cowpox virus	GRI-90	CPXV-GRI	X94355
Orthopoxvirus	Ectromelia virus	Moscow	ECTV-Mos	NC_004105
Orthopoxvirus	Ectromelia virus	Naval	ECTV-Nav	n/a
Orthopoxvirus	Monkeypox virus	Congo_2003_358	MPXV-COG_2003_358	DQ011154
Orthopoxvirus	Monkeypox virus	COP-58	MPXV-COP58	AY753185
Orthopoxvirus	Monkeypox virus	Liberia_1970_184	MPXV-LBR_1970_184	DQ011156
Orthopoxvirus	Monkeypox virus	Sierra Leone	MPXV-SLE	AY741551
Orthopoxvirus	Monkeypox virus	USA_2003_039	MPXV-USA_2003_039	DQ011157
Orthopoxvirus	Monkeypox virus	USA_2003_044	MPXV-USA_2003_044	DQ011153
Orthopoxvirus	Monkeypox virus	Walter Reed 267	MPXV-WR267	AY603973
Orthopoxvirus	Monkeypox virus	Zaire	MPXV-ZAR	NC_003310
Orthopoxvirus	Monkeypox virus	Zaire_1979-005	MPXV-ZAR_1979_005	DQ011155
Orthopoxvirus	Taterapox virus	Dahomey 1968	TATV-DAH68	NC_008291
Orthopoxvirus	Vaccinia virus	3737	VACV-3737	DQ377945
Orthopoxvirus	Vaccinia virus	Acambis 2000	VACV-Acam2000	AY313847

Genus	Species	Strain	Abbreviation	GenBank Accession
Orthopoxvirus	Vaccinia virus	Acambis 3	VACV-Acam3	AY313848
Orthopoxvirus	Vaccinia virus	Acambis 3000 Modified Virus Ankara	VACV-Acam3000	AY603355
Orthopoxvirus	Vaccinia virus	AGR-MVA-572pre	VACV-AGR_MVA_572pre	DQ983239
Orthopoxvirus	Vaccinia virus	chorioallantois vaccinia virus Ankara (CVA)	VACV-CVA	AM501482
Orthopoxvirus	Vaccinia virus	Copenhagen	VACV-Cop	M35027
Orthopoxvirus	Vaccinia virus	DUKE	VACV-DUKE	DQ439815
Orthopoxvirus	Vaccinia virus	LC16m8	VACV-LC16m8	AY678275
Orthopoxvirus	Vaccinia virus	LC16mO	VACV-LC16mO	AY678277
Orthopoxvirus	Vaccinia virus	Lister	VACV-Lister	AY678276
Orthopoxvirus	Vaccinia virus	Lister_VACV107	VACV-Lister_VACV107	DQ121394
Orthopoxvirus	Vaccinia virus	MNR-76	HSPV-MNR76	DQ792504
Orthopoxvirus	Vaccinia virus	MVA-I721	VACV-MVA_I721	DQ983236
Orthopoxvirus	Vaccinia virus	recombinant GLV-1h68	VACV-REC_GLV_1h68	EU410304
Orthopoxvirus	Vaccinia virus	Utrecht	RPXV-Utr	AY484669
Orthopoxvirus	Vaccinia virus	Western Reserve	VACV-WR	NC_006998
Orthopoxvirus	Variola virus	Afghanistan 1970 Variolator 4	VARV-AFG70	DQ437580
Orthopoxvirus	Variola virus	Bangladesh 1974 (nur islam)	VARV-BGD74_nur	DQ441420
Orthopoxvirus	Variola virus	Bangladesh 1974 (Shahzaman)	VARV-BGD74_shz	DQ441421
Orthopoxvirus	Variola virus	Bangladesh 1974 (Solaiman)	VARV-BGD74_sol	DQ441422
Orthopoxvirus	Variola virus	Bangladesh 1975	VARV-BGD75maj	L22579
Orthopoxvirus	Variola virus	Bangladesh 1975 v75-550 Banu	VARV-BGD75_Banu	DQ437581
Orthopoxvirus	Variola virus	Benin, Dahomey 1968 (v68-59)	VARV-BEN68	DQ441416
Orthopoxvirus	Variola virus	Botswana 1972 (v72-143)	VARV-BWA72	DQ441417
Orthopoxvirus	Variola virus	Botswana 1973 (v73-225)	VARV-BWA73	DQ441418
Orthopoxvirus	Variola virus	Brazil 1966 (v66-39 Sao Paulo)	VARV-BRA66	DQ441419
Orthopoxvirus	Variola virus	China Horn 1948 Sabin Lab July 1948	VARV-CHN48	DQ437582

Genus	Species	Strain	Abbreviation	GenBank Accession
Orthopoxvirus	Variola virus	Congo 1970 v70-46 Kinshasa	VARV-COG70_46	DQ437583
Orthopoxvirus	Variola virus	Congo 9 1970 (v74-227 Gispen)	VARV-COG70_227	DQ441423
Orthopoxvirus	Variola virus	Ethiopia 1972 (Eth16 R14-1X-72 Addis)	VARV-ETH72_16	DQ441424
Orthopoxvirus	Variola virus	Ethiopia 1972 (Eth17 R14-1X-72 Addis)	VARV-ETH72_17	DQ441425
Orthopoxvirus	Variola virus	Garcia 1966	VARV-Gar_1966	Y16780
Orthopoxvirus	Variola virus	Germany 1958 Heidelberg	VARV-DEU58	DQ437584
Orthopoxvirus	Variola virus	Guinea 1969 (005)	VARV-GIN69	DQ441426
Orthopoxvirus	Variola virus	India 1953 (Kali-Muthu-M50 Madras)	VARV-IND53_mad	DQ441427
Orthopoxvirus	Variola virus	India 1953 (New Delhi)	VARV-IND53_ndel	DQ441428
Orthopoxvirus	Variola virus	India 1964 7124 Vellore	VARV-IND64_vel4	DQ437585
Orthopoxvirus	Variola virus	India 1964 7125 Vellore	VARV-IND64_vel5	DQ437586
Orthopoxvirus	Variola virus	India 3 Major 1967	VARV-IND3_1967	NC_001611
Orthopoxvirus	Variola virus	Iran 1972 2602 Tabriz	VARV-IRN72	DQ437587
Orthopoxvirus	Variola virus	Japan 1946 (Yamada MS-2(A) Tokyo)	VARV-JPN46_yam	DQ441429
Orthopoxvirus	Variola virus	Japan 1951 (Harper, Masterseed)	VARV-JPN51_hrpr	DQ441430
Orthopoxvirus	Variola virus	Japan 1951 (Stillwell, Masterseed)	VARV-JPN51_stwl	DQ441431
Orthopoxvirus	Variola virus	Korea 1947 (Lee, Masterseed)	VARV-KOR47	DQ441432
Orthopoxvirus	Variola virus	Kuwait 1967 (K1629)	VARV-KWT67	DQ441433
Orthopoxvirus	Variola virus	Nepal 1973 V73-175	VARV-NPL73	DQ437588
Orthopoxvirus	Variola virus	Niger 1969 (001, importation from Nigeria)	VARV-NER69	DQ441434
Orthopoxvirus	Variola virus	Pakistan 1969 (Rafig Lahore)	VARV-PAK_1969	DQ437589
Orthopoxvirus	Variola virus	Sierra Leone 1969 (V68-258)	VARV-SLE68	DQ441437
Orthopoxvirus	Variola virus	Somalia 1977 (V77-1252)	VARV-SOM77_1252	DQ441438
Orthopoxvirus	Variola virus	Somalia 1977 (V77-1605)	VARV-SOM77_1605	DQ441439
Orthopoxvirus	Variola virus	Somalia 1977 V77-2479	VARV-SOM77_ali	DQ437590

Genus	Species	Strain	Abbreviation	GenBank Accession
Orthopoxvirus	Variola virus	South Africa 1965 (102 Natal, Ingwavuma)	VARV-ZAF65_102	DQ441435
Orthopoxvirus	Variola virus	South Africa 1965 (103 Tvaal, Nelspruit)	VARV-ZAF65_103	DQ441436
Orthopoxvirus	Variola virus	Sudan 1947 (Juba)	VARV-SDN47_jub	DQ441440
Orthopoxvirus	Variola virus	Sudan 1947 (Rumbec)	VARV-SDN47_rum	DQ441441
Orthopoxvirus	Variola virus	Sumatra 1970 V70-222	VARV-SUM70_222	DQ437591
Orthopoxvirus	Variola virus	Sumatra 1970 V70-228	VARV-SUM70_228	DQ441442
Orthopoxvirus	Variola virus	Syria 1972 V72-199	VARV-SYR72	DQ437592
Orthopoxvirus	Variola virus	Tanzania 1965 kembula	VARV-TZA65	DQ441443
Orthopoxvirus	Variola virus	United Kingdom 1946 Harvey	VARV-GBR44_harv	DQ441444
Orthopoxvirus	Variola virus	United Kingdom 1946 Hinden (Middlesex)	VARV-GBR46_hind	DQ441445
Orthopoxvirus	Variola virus	United Kingdom 1947 Higgins (Staffordshire)	VARV-GBR47_hig	DQ441446
Orthopoxvirus	Variola virus	United Kingdom 1952 Butler	VARV-GBR52_but	DQ441447
Orthopoxvirus	Variola virus	Yugoslavia 1972 V72-164	VARV-YUG72	DQ441448
Suipoxvirus	Swinepox virus	Nebraska 17077-99	SWPV-Neb	NC_003389
Suipoxvirus	Swinepox virus	Nebraska 17077-99	SWPV-Neb	NC_003389
Unclassified Poxviridae	Mule deer poxvirus	W-1170-84	DPV-W1170_84	AY689437
Unclassified Poxviridae	Mule deer poxvirus	W-848-83	DPV-W848_83	NC_006966
Yatapoxvirus	Tanapox virus	Davis	YLDV-Davis	NC_002642
Yatapoxvirus	Tanapox virus	isolate TPV-RoC	TANV-COD	EF420157
Yatapoxvirus	Tanapox virus	Kenya	TANV-KEN	NC_009888
Yatapoxvirus	Tanapox virus	Zimbabwe	CRV-ZWE	NC_008030
Yatapoxvirus	Yaba monkey tumor virus	Amano	YMTV-Amano	NC_005179
