# Supplementary material for: Genes in the terminal regions of orthopoxvirus genomes experience adaptive molecular evolution
Source: BMC Genomics. 2011 May 23;12:261. doi: 10.1186/1471-2164-12-261 (PMC3123329; doi:10.1186/1471-2164-12-261)
Supplement: Additional File 2 — Gene families excluded from analysis. [file 1471-2164-12-261-S2.PDF]

Gene families excluded from analysis

ORFnumber	Gene Family	Reason Excluded
CPXV-BR-001/235	CPV-B-001	Duplicated
CPXV-BR-002/234	CPV-B-002	Duplicated
CPXV-BR-003/233	Chemokine binding protein	Duplicated
CPXV-BR-004	CPV-B-004	Small Family
CPXV-BR-005/014/232	TNF receptor (CrmB)	Duplicated
CPXV-BR-006/226/231	Ankyrin (Cop-B25R)	Duplicated
CPXV-BR-007/230	CPV-B-007	Duplicated
CPXV-BR-008/229	Ankyrin (CPXV-008)	Duplicated
CPXV-BR-009/214/228	Unknown (Cop-B22R)	Duplicated
CPXV-BR-010/038	Alpha-amanitin sensitivity	Duplicated
CPXV-BR-011/219	Ankyrin (Bang-B18R)	Duplicated
CPXV-BR-012	CPV-B-012	Small Family
CPXV-BR-013/199	Kelch-like (Cop-A55R)	Duplicated
CPXV-BR-015	Unknown (CPXV-GRI-D13L)	Pseudogene
CPXV-BR-016/019/217	Ankyrin (Cop-B18R)	Duplicated
CPXV-BR-022/033	IL-1 Receptor antagonist	Duplicated
CPXV-BR-031/032	Unknown (Cop-C5L)	Duplicated
CPXV-BR-042/213/223	Serpin 1,2,3	Duplicated
CPXV-BR-047	CPV-B-047	Small Family
CPXV-BR-052	VV_Cop-F ORF B	Small Family
CPXV-BR-059	VV_Cop-F ORF D	Small Family
CPXV-BR-064	CPV-B-063	Small Family
CPXV-BR-081	CPV-B-078A	Small Family
CPXV-BR-099	VV_Cop-G ORF B	Small Family
CPXV-BR-119	CPV-B-116	Small Family
CPXV-BR-123	VV_Cop-D ORF B	Small Family
CPXV-BR-134	VV_Tan-unknown-16	Small Family
CPXV-BR-157	CPV-B-152A	Small Family
CPXV-BR-164/166	P4c precursor	Duplicated
CPXV-BR-165	Unknown (CPV-B-160)	Small Family
CPXV-BR-176	VV_Cop-A ORF M	Small Family
CPXV-BR-197	TNF receptor (CrmC)	Pseudogene
CPXV-BR-198	CPV-B-192	Small Family
CPXV-BR-220	CPV-B-214	Small Family
CPXV-BR-221	kelch-like (EV-M-167)	Difficult Alignment
CPXV-BR-222	CPV-B-216	Small Family
CPXV-BR-163	A-type Inclusion	Difficult Alignment, Computational Time
CPXV-BR-225	Surface Glycoprotein	Computational Time
