# Supplementary material for: Genes in the terminal regions of orthopoxvirus genomes experience adaptive molecular evolution
Source: BMC Genomics. 2011 May 23;12:261. doi: 10.1186/1471-2164-12-261 (PMC3123329; doi:10.1186/1471-2164-12-261)
Supplement: Additional File 3 — Basic statistics of genes used in analysis. [file 1471-2164-12-261-S3.PDF]

Basic statistics of gene families used in analysis

ORF Number	Family Name	# Sequences	Treelength M2a	k M2a	Treelength M8	k M8	Significant
CPXV-BR-227	TNF_receptor_(CrmD)	6	0.225	1.929	0.226	1.897	No
CPXV-BR-017	Ankyrin_(CPXV_017)	11	0.246	5.864	0.239	5.695	No
CPXV-BR-082	Glutaredoxin_1_(Cop_O2L)	15	0.269	2.493	0.268	2.470	No
CPXV-BR-046	Unknown_(Cop_K7R)	22	0.350	5.420	0.338	5.543	M2a & M8
CPXV-BR-190	IL_1_signaling_inhibitor_(Cop_A46R)	29	0.406	6.150	0.376	5.919	No
CPXV-BR-207	Virulence_ER_resident	14	0.406	3.588	0.400	3.590	No
CPXV-BR-180	Unknown_(Gar_A43R)	13	0.423	6.568	0.419	6.571	No
CPXV-BR-192	Thymidylate_kinase	26	0.435	2.911	0.407	2.711	M2a & M8
CPXV-BR-183	Lectin_homolog	18	0.501	5.286	0.488	5.211	No
CPXV-BR-036	Unknown_(Cop_C1L)	36	0.543	5.443	0.458	4.558	M2a & M8
CPXV-BR-055	Unknown_(Cop_F7L)	15	0.562	4.556	0.500	3.663	M2a & M8
CPXV-BR-204	Ankyrin_(Cop_B4R)	36	0.568	5.059	0.538	4.900	M2a & M8
CPXV-BR-026	Unknown_(Tan_TC10L)	16	0.576	3.784	0.572	3.791	No
CPXV-BR-020	Unknown_(Bang_D3L)	14	0.604	2.536	0.592	2.339	No
CPXV-BR-178	IEV_specific_(Cop_A36R)	25	0.620	3.163	0.614	3.162	No
CPXV-BR-184	Virulence__Secreted_(Cop_A41L)	25	0.626	4.381	0.622	4.286	No
CPXV-BR-030	Unknown_(Cop_C6L)	24	0.650	4.144	0.643	4.027	No
CPXV-BR-039	Ankyrin_(Cop_M1L)	42	0.660	3.583	0.638	3.524	No
CPXV-BR-193	Putative_Phosphotransferase_anion transport_protein_(Cop_A49R)	22	0.661	4.254	0.651	4.133	No
CPXV-BR-034	Complement_binding_(secreted)	26	0.678	2.994	0.660	2.894	No
CPXV-BR-054	Unknown_(Cop_F6L)	14	0.705	2.299	0.690	1.970	M2a & M8
CPXV-BR-185	Profilin_homolog_(Cop_A42R)	21	0.756	2.652	0.743	2.561	No
CPXV-BR-053	36kDa_major_membrane_protein (Cop_F5L)	34	0.771	3.648	0.755	3.548	M8
CPXV-BR-191	Unknown_(Cop_A47L)	30	0.788	4.515	0.751	3.857	M2a & M8
CPXV-BR-187	Unknown_(MVA_156R)	12	0.802	4.262	0.743	3.887	No
CPXV-BR-035	Kelch_like_(Cop_C2L)	55	0.805	5.825	0.770	5.552	M2a & M8
CPXV-BR-048	Apoptosis_inhibitor_(mitochondrial associated)	52	0.809	5.125	0.766	4.512	M2a & M8

ORF Number	Family Name	# Sequences	Treelength M2a	k M2a	Treelength M8	k M8	Significant
CPXV-BR-216	Unknown_(Cop_B17L)	29	0.817	4.230	0.802	4.103	M2a & M8
CPXV-BR-206	Unknown_(Cop_B6R)	25	0.880	4.630	0.826	4.503	No
CPXV-BR-028	Unknown_(Cop_C8L)	16	0.905	2.251	0.880	2.002	No
CPXV-BR-041	Ankyrin_NFkB_inhib_(Cop_K1L)	30	0.940	3.738	0.918	3.553	No
CPXV-BR-211	Unknown_(Cop_B11R)	21	0.945	5.231	0.937	5.099	No
CPXV-BR-210	Kelch_like_(CPV_GRI_B9R)	12	1.027	2.822	1.029	2.823	No
CPXV-BR-212	Ser_Thr_Kinase_(Cop_B12R)	32	1.080	2.980	0.978	2.701	M2a & M8
CPXV-BR-024	IL_18_BP_(Bsh_D7L)	35	1.110	3.274	1.060	2.974	M2a & M8
CPXV-BR-203	Schlafen_(Cop_B2R)	39	1.139	3.904	1.099	3.569	M2a & M8
CPXV-BR-201	Guanylate_kinase	33	1.195	3.860	1.178	3.738	No
CPXV-BR-182	Semaphorin	41	1.287	3.277	1.063	2.975	M2a & M8
CPXV-BR-186	Membrane_glycoprotein_class_I	25	1.386	3.291	1.327	3.081	M8
CPXV-BR-018	MPV_Z_N3R	6	1.658	2.981	1.341	2.683	No
CPXV-BR-027	Ankyrin_(Cop_C9L)	47	1.811	2.911	1.729	2.750	M8
CPXV-BR-025	Ankyrin_Host_Range_(Bang_D8L)	55	2.108	3.510	1.861	3.419	M8
CPXV-BR-215	IL_1_beta_receptor	42	3.193	4.349	2.833	4.153	M2a & M8
CPXV-BR-224	Unknown_(Cop_C14L)	18	3.510	2.202	3.103	2.180	No
CPXV-BR-086	Ribonucleotide_Reductase_large_subunit	42	6.468	3.082	5.091	2.967	M8
CPXV-BR-044	Nicking_Joining_Enzyme_(Cop_K4L)	26	6.489	2.318	5.579	2.346	M8
CPXV-BR-060	Unknown_(Cop_F11L)	35	7.910	2.666	7.512	2.507	No
CPXV-BR-084	Unknown_(Cop_I2L)	25	7.940	2.856	8.470	2.840	M8
CPXV-BR-151	IMV_MP_Virulence_factor_(Cop_A14.5L)	16	8.199	4.088	8.562	4.163	No
CPXV-BR-045	Putative_monoglyceride_lipase	29	8.446	3.750	8.040	4.002	No
CPXV-BR-078	S_S_formation_pathway_(Cop_E10R)	34	8.465	3.166	9.079	2.846	No
CPXV-BR-138	Thioredoxin_like_(Cop_A2.5L)	27	8.645	2.446	8.847	2.119	No
CPXV-BR-079	Virion_core_protein_(Cop_E11L)	22	8.725	3.117	7.543	2.975	M8
CPXV-BR-109	Poly(A)_polymerase_small_(VP39)	47	8.956	2.745	8.664	2.427	No
CPXV-BR-168	IMV_MP_Virus_entry_(Cop_A28L)	31	9.794	3.229	10.602	2.807	No
CPXV-BR-188	Hydroxysteroid_dehydrogenase	49	9.960	2.371	10.177	2.200	M8
CPXV-BR-137	VLTF_3_(late_transcription_factor_3)	36	9.997	2.592	11.893	2.141	No
CPXV-BR-209	Virulence_factor_(Cop_B9R)	29	10.110	3.371	9.653	3.223	No
CPXV-BR-158	Entry_and_Cell_Cell_Fusion_(Cop_A21L)	34	10.169	3.734	11.374	3.607	No
CPXV-BR-106	Entry_and_Fusion_IMV_protein_(Cop_L5R)	27	10.204	2.674	10.724	2.496	No

ORF Number	Family Name	# Sequences	Treelength M2a	k M2a	Treelength M8	k M8	Significant
CPXV-BR-122	Virion_Core_(Cop_D2L)	26	10.486	2.955	10.486	2.837	No
CPXV-BR-107	Virion_morph_(Cop_J1R)	34	10.646	2.348	11.449	2.057	No
CPXV-BR-149	Virion_Maturation_(Cop_A13L)	33	10.647	2.699	10.982	2.511	No
CPXV-BR-040	NFkB_inh_(Cop_M2L)	31	10.660	2.681	9.712	2.577	No
CPXV-BR-092	Unknown_(Cop_G3L)	26	11.023	2.940	12.878	2.768	No
CPXV-BR-076	ER_localized_MP(Cop_E8R)	42	11.110	2.458	11.427	2.197	M8
CPXV-BR-156	Unknown_(Cop_A19L)	26	11.179	3.139	10.809	2.839	M8
CPXV-BR-077	DNA_pol	53	11.191	2.650	11.539	2.266	No
CPXV-BR-136	VLTF_2_(late_transcription_factor_2)	34	11.385	2.811	12.092	2.753	No
CPXV-BR-096	RNA_pol_(RPO7)	22	11.462	1.949	14.471	1.955	No
CPXV-BR-110	RNA_pol_(RPO22)	28	11.517	3.754	12.062	3.268	No
CPXV-BR-102	Myristylated_MP_IMV_(Cop_L1R)	35	11.634	2.175	11.857	2.177	No
CPXV-BR-170	Virion_Morph_(Cop_A30L)	23	11.639	2.908	14.044	2.825	No
CPXV-BR-152	Unknown_(Cop_A15L)	24	11.741	2.886	15.733	2.958	No
CPXV-BR-112	RNA_pol_(RPO147)	59	11.747	2.686	nd	nd	No
CPXV-BR-129	Carbonic_anhydrase_Virion	29	11.747	3.385	8.121	3.310	M2a & M8
CPXV-BR-125	Uracil_DNA_glycosylase	38	11.843	2.891	12.261	2.625	No
CPXV-BR-130	mutT_motif_NTP_PPH	30	11.844	3.158	13.336	2.641	No
CPXV-BR-169	RNA_pol_35(RPO35)	37	11.886	2.367	12.975	2.169	M8
CPXV-BR-037	Virokine_NFkB_inh_Str_resemblence_to apoptotic_reg_(Cop_N1L)	25	11.999	3.187	11.526	3.171	No
CPXV-BR-068	DNA_binding_phosphoprotein_(Cop_F17R)	35	12.113	2.223	12.894	2.187	No
CPXV-BR-062	Phospholipase_EEV_(Cop_F13L)	47	12.215	2.309	12.300	2.267	No
CPXV-BR-162	RNA_pol_132(RPO132)	50	12.452	2.965	11.968	2.514	M2a & M8
CPXV-BR-113	Tyr_Ser_phosphatase	33	12.749	3.029	14.214	2.526	No
CPXV-BR-075	Soluble_Myristyl_EEV_(Cop_E7R)	27	12.917	3.222	11.511	3.160	No
CPXV-BR-063	Unknown_(Cop_F14L)	19	12.960	3.715	8.643	2.674	M2a & M8
CPXV-BR-093	VLTF_(late_transcription_elongation_factor_ Cop_G2R)	38	12.972	2.448	12.876	2.330	No
CPXV-BR-143	VETF_L_(early_transcription_factor_large)	45	13.024	2.979	13.111	2.575	No
CPXV-BR-154	IMV_MP_PO4_(Cop_A17L)	34	13.111	2.514	12.080	2.281	M8
CPXV-BR-194	DNA_ligase	44	13.208	2.231	12.986	2.008	No
CPXV-BR-111	Unknown_MP_(Cop_J5L)	29	13.292	2.361	13.660	2.215	No

ORF Number	Family Name	# Sequences	Treelength M2a	k M2a	Treelength M8	k M8	Significant
CPXV-BR-073	Virosome_component	44	13.459	2.956	13.614	2.886	M8
CPXV-BR-155	DNA_Helicase_transcription	48	13.549	2.869	13.847	2.678	M8
CPXV-BR-135	Trimeric_virion_coat_protein (rifampicin_res)	43	13.627	2.749	14.032	2.587	No
CPXV-BR-021	EGF_Growth_factor	37	13.722	3.624	13.268	3.355	M2a & M8
CPXV-BR-133	Small_capping_enzyme	33	13.825	2.719	14.702	2.443	No
CPXV-BR-090	RNA_helicase_NPH_II	50	13.875	2.628	14.165	2.440	M8
CPXV-BR-108	Thymidine_kinase	35	13.885	2.465	16.431	2.342	No
CPXV-BR-132	NPH_I_Helicase_virion	46	13.925	2.598	15.597	2.376	M8
CPXV-BR-100	VLTF_1_(Cop_G8R)	38	14.071	2.857	14.836	2.945	No
CPXV-BR-097	Unknown_(Cop_G6R)	43	14.108	3.007	13.480	2.889	No
CPXV-BR-058	Ser_Thr_kinase_Morph_(Cop_F10L)	47	14.116	2.843	13.861	2.605	M8
CPXV-BR-065	Unknown(YMTV_28.5L)	19	14.249	3.648	13.053	3.594	No
CPXV-BR-074	Unknown_(Cop_E6R)	44	14.267	2.648	15.273	2.360	M8
CPXV-BR-124	Virion_core_(Cop_D3R)	42	14.371	3.149	15.114	2.841	M8
CPXV-BR-083	DNA_binding_protein_(Cop_I1L)	44	14.622	3.114	14.857	2.780	M8
CPXV-BR-103	Unknown_(Cop_L2R)	28	14.629	2.618	13.918	2.821	M8
CPXV-BR-072	RNA_pol_(RPO30)	39	14.748	2.953	13.174	2.623	M8
CPXV-BR-171	Unknown_(YMTV_120.5L)	17	14.787	3.751	13.416	4.170	M2a & M8
CPXV-BR-148	Structural_protein_(Cop_A12L)	39	14.847	2.073	13.807	2.039	No
CPXV-BR-200	Hemagglutinin	50	14.913	3.533	13.501	3.413	M2a & M8
CPXV-BR-104	Internal_Virion_Protein_(Cop_L3L)	45	15.053	2.832	15.124	2.651	M8
CPXV-BR-043	IFN_resistance_eIF2_alpha_like_PKR inhibitor	31	15.233	2.695	15.002	2.722	No
CPXV-BR-091	Predicted_metallo_protease_(Cop_G1L)	50	15.241	2.814	15.317	2.568	No
CPXV-BR-128	RNA_pol_18(RPO18)	31	15.244	3.173	14.754	2.907	No
CPXV-BR-121	Large_capping_enzyme	55	15.324	2.418	15.066	2.214	M8
CPXV-BR-127	Morph_VETF_s_early_transcription_factor small	45	15.337	3.112	15.189	2.706	M8
CPXV-BR-095	Unknown_(Cop_G5R)	46	15.416	2.865	16.807	2.491	M8
CPXV-BR-116	RAP94_(RNA_pol_assoc_protein)	53	15.448	3.257	14.706	2.695	M8
CPXV-BR-101	Entry_fusion_complex_protein	41	15.508	2.463	16.258	2.247	No

ORF Number	Family Name	# Sequences	Treelength M2a	k M2a	Treelength M8	k M8	Significant
CPXV-BR-175	C_type_lectin_like_EEV_protein_(Cop_A34R)	32	15.518	3.076	16.050	3.204	No
CPXV-BR-089	Virion_Core_Protease	44	15.530	3.250	15.896	2.919	No
CPXV-BR-141	RNA_pol_19_(RPO19)	29	15.652	2.078	15.656	2.037	No
CPXV-BR-118	Topoisomerase_type_I	40	15.726	3.348	18.674	3.047	No
CPXV-BR-159	DNA_Processivity_factor	43	15.810	2.910	16.406	2.557	M8
CPXV-BR-114	Entry_and_Cell_Cell_Fusion_(Cop_H2R)	35	15.959	2.716	16.430	2.690	No
CPXV-BR-061	IEV_associated_(Cop_F12L)	53	16.130	2.984	16.995	2.869	M8
CPXV-BR-057	S_S_bond_formation_pathway_protein (Cop_F9L)	36	16.143	2.909	15.500	2.887	No
CPXV-BR-146	P4a_precursor	57	16.257	2.465	17.852	2.270	M8
CPXV-BR-147	Viral_membrane_formation_(Cop_A11R)	39	16.410	2.580	16.445	2.505	No
CPXV-BR-218	IFN_alpha_beta_receptor	43	16.416	2.995	15.824	2.676	No
CPXV-BR-153	Myristylated_Entry_Cell_cell_fusion_protein_ (Cop_A16L)	46	16.508	2.570	16.400	2.525	No
CPXV-BR-196	Intracellular_TLR_and_IL_1_signaling inhibitor_(Cop_A52R)	29	16.511	2.166	15.130	2.214	M8
CPXV-BR-070	Unknown_(Cop_E2L)	48	16.523	2.881	16.296	2.686	M8
CPXV-BR-140	Core_protein_(Cop_A4L)	41	17.005	3.307	14.873	3.107	M2a & M8
CPXV-BR-181	CD47_like	41	17.055	3.352	18.695	3.314	No
CPXV-BR-208	IFN_gamma_receptor	38	17.109	2.693	16.009	2.577	M8
CPXV-BR-167	Fusion_protein_(Cop_A27L)	33	17.267	2.456	16.153	2.431	No
CPXV-BR-071	IFN_resistance_PKR_inhibitor (Z_DNA_binding)	41	17.328	2.434	17.898	2.473	M8
CPXV-BR-126	NTPase_DNA_replication	50	17.456	2.675	17.507	2.501	M8
CPXV-BR-115	IMV_heparin_binding_surface_protein	41	17.748	2.652	17.042	2.547	M8
CPXV-BR-067	Unknown_(Cop_F16L)	43	17.787	3.536	18.103	3.264	No
CPXV-BR-195	Unknown_(Cop_A51R)	51	18.125	3.040	17.992	2.978	M8
CPXV-BR-205	Complement_control_CD46_EEV	47	18.394	3.606	16.959	3.370	M8
CPXV-BR-160	Holliday_junction_resolvase	35	18.481	3.344	15.799	3.086	M8
CPXV-BR-202	Ser_Thr_Kinase_(Cop_B1R)	41	18.512	2.880	20.485	2.672	No
CPXV-BR-120	Unknown_(Cop_H7R)	38	18.843	2.594	19.586	2.627	M8
CPXV-BR-085	DNA_binding_phosphoprotein_(Cop_I3L)	38	18.886	2.617	18.953	2.420	M8

ORF Number	Family Name	# Sequences	Treelength M2a	k M2a	Treelength M8	k M8	Significant
CPXV-BR-145	Membrane_protein_(Cop_A9L)	34	19.045	4.087	10.722	3.610	M2a & M8
CPXV-BR-150	IMV_PO4_MP_(Cop_A14L)	32	19.052	3.127	26.861	3.194	No
CPXV-BR-173	ATPase_DNA_packaging_protein	36	19.273	2.891	20.622	2.685	No
CPXV-BR-080	Unknown_(Cop_O1L)	59	19.485	2.675	18.702	2.487	M8
CPXV-BR-144	VITF_3_34kda_subunit_(Cop_A8R)	35	19.841	2.715	17.972	2.424	M8
CPXV-BR-049	dUTPase	31	19.862	2.442	19.821	2.362	No
CPXV-BR-098	Virion_assembly_protein_(Cop_G7L)	42	19.909	2.797	20.267	2.448	M8
CPXV-BR-174	EEV_Glycoprotein_(Cop_A33R)	38	20.002	2.526	18.238	2.452	No
CPXV-BR-177	Unknown_(Cop_A35R)	32	20.089	2.931	22.064	2.844	No
CPXV-BR-088	Telomere_Binding_protein	46	20.448	2.817	22.071	2.504	No
CPXV-BR-142	Virion_Morphogenesis_(Cop_A6L)	43	20.528	2.944	21.220	2.727	M8
CPXV-BR-094	Glutaredoxin_2_(Cop_G4L)	31	20.935	2.319	21.005	2.199	No
CPXV-BR-131	mutT_motif_NPH_PPH_RNA_levels_regulator	36	21.310	2.490	20.489	2.592	M8
CPXV-BR-029	Host_range_virulence_factor	38	21.607	2.946	24.429	2.835	No
CPXV-BR-069	Poly_(A)_polymerase_large_(VP55)	37	21.630	3.112	21.126	2.918	M8
CPXV-BR-179	Unknown_(Cop_A37R)	38	22.907	4.077	25.085	4.115	M8
CPXV-BR-066	Unknown_Conserved_(Cop_F15L)	29	23.355	3.806	19.693	3.484	M8
CPXV-BR-161	VITF_3_45kda_subunit_(Cop_A23R)	39	23.374	3.085	23.911	2.916	No
CPXV-BR-056	Cytoplasmic_protein_(Cop_F8L)	34	24.268	4.674	15.053	3.826	M2a & M8
CPXV-BR-023	Ubiquitin_Ligase_Host_defense_modulator	50	24.573	3.044	25.779	2.842	M8
CPXV-BR-117	VLTF_4_(late_transcription_factor_4)	42	24.581	2.728	21.355	2.458	M8
CPXV-BR-050	Kelch_like_(Cop_F3L)	69	27.431	2.696	29.868	2.691	M8
CPXV-BR-105	Core_package_transcription	37	28.490	2.830	23.923	2.604	M8
CPXV-BR-087	IMV_protein_VP13	31	31.073	2.311	30.898	2.346	No
CPXV-BR-189	Superoxide_dismutase_like	33	32.686	3.002	23.440	3.188	No
CPXV-BR-172	Unknown_(Cop_A31R)	27	43.020	4.865	17.725	4.308	M2a & M8
