# Supplementary material for: Genes in the terminal regions of orthopoxvirus genomes experience adaptive molecular evolution
Source: BMC Genomics. 2011 May 23;12:261. doi: 10.1186/1471-2164-12-261 (PMC3123329; doi:10.1186/1471-2164-12-261)
Supplement: Additional File 4 — Genes under diversifying selection identified by model M8 only. [file 1471-2164-12-261-S4.PDF]

ORF Number	Gene Family Name	Category	VACV-Cop ORF	Specific Function	Encapsidated
CPXV-BR-023	Ubiquitin_Ligase_Host_defense_ modulator	Host Response Modifier	n/a	Ubiquitin ligase activity	No
CPXV-BR-025	Ankyrin_Host_Range (Bang_D8L)	Host Response Modifier	n/a	Binds and inactivates NFkB	No
CPXV-BR-027	Ankyrin_(Cop_C9L)	Unknown	C9L	Unknown	No
CPXV-BR-044	Nicking_Joining_Enzyme (Cop_K4L)	DNA metabolism	K4L	Nicks and joins DNA at telomeres	Yes
CPXV-BR-050	Kelch_like_(Cop_F3L)	Host Response Modifier	F3L	Modulation of inflammatory response	No
CPXV-BR-053	36kDa_major_membrane_protein _(Cop_F5L)	Unknown	F5L	Membrane protein unknown function	No
CPXV-BR-058	Ser_Thr_kinase_Morph (Cop_F10L)	Structure/Assembly	F10L	Membrane remodeling for morphogenesis	Yes
CPXV-BR-061	IEV_associated_(Cop_F12L)	Structure/Assembly	F12L	Transport of IEV to cell surface	No
CPXV-BR-066	Unknown_Conserved (Cop_F15L)	Unknown	F15L	Unknown	No
CPXV-BR-069	Poly_(A)_polymerase_large (VP55)	DNA metabolism	E1L	adds poly-A tail to viral transcripts	Yes
CPXV-BR-070	Unknown_(Cop_E2L)	Structure/Assembly	E2L	IEV morphogenesis	No
CPXV-BR-071	IFN_resistance_PKR_inhibitor (Z_DNA_binding)	Host Response Modifier	E3L	inhibitor of cellular dsRNA response	Yes
CPXV-BR-072	RNA_pol_(RPO30)	RNA metabolism	E4L	RNA polymerase subunit	Yes
CPXV-BR-073	Virosome_component	Unknown	E5R	Unknown - found in virosome	No
CPXV-BR-074	Unknown_(Cop_E6R)	Structure/Assembly	E6R	Essential for virion morphogenesis	Yes
CPXV-BR-076	ER_localized_MP(Cop_E8R)	DNA metabolism	E8R	localized to membranes that enclose sites of DNA replication	Yes

ORF Number	Gene Family Name	Category	VACV-Cop ORF	Specific Function	Encapsidated
CPXV-BR-079	Virion_core_protein_(Cop_E11L)	Structure/Assembly	E11L	Virion component	Yes
CPXV-BR-080	Unknown_(Cop_O1L)	Unknown	O1L	Unknown	No
CPXV-BR-083	DNA_binding_protein_(Cop_I1L)	DNA metabolism	I1L	Binds telomeres in IMV and target of immune response	Yes
CPXV-BR-084	Unknown_(Cop_I2L)	Entry/Exit	I2L	Viral entry	Yes
CPXV-BR-085	DNA_binding_phosphoprotein (Cop_I3L)	DNA metabolism	I3L	essential ssDN binding involved in DNA replication or repair	Yes
CPXV-BR-086	Ribonucleotide_Reductase_large_ subunit	RNA metabolism	I4L	makes dNTPs, increases virulence when inoculated in non-replicating cells	No
CPXV-BR-090	RNA_helicase_NPH_II	RNA metabolism	I8R	RNA helicase necessary for transcription	Yes
CPXV-BR-095	Unknown_(Cop_G5R)	Structure/Assembly	G5R	Morphogenesis	Yes
CPXV-BR-098	Virion_assembly_protein (Cop_G7L)	Structure/Assembly	G7L	uptake of virosome by viral crescents to form IV	Yes
CPXV-BR-103	Unknown_(Cop_L2R)	Unknown	L2R	Unknown	No
CPXV-BR-104	Internal_Virion_Protein (Cop_L3L)	RNA metabolism	L3L	early gene transcription in virion	Yes
CPXV-BR-105	Core_package_transcription	RNA metabolism	L4R	ssRNA/ssDNA binding protein involved in early mRNA transcription	Yes
CPXV-BR-115	IMV_heparin_binding_surface protein	Structure/Assembly	H3L	helps form mature virions, target of neutralizing antibodies	Yes
CPXV-BR-116	RAP94_(RNA_pol_assoc_protein)	DNA metabolism	H4L	early stage transcription factor	Yes
CPXV-BR-117	VLTF_4_(late_transcription factor_4)	RNA metabolism	H5R	transcription factor	Yes
CPXV-BR-120	Unknown_(Cop_H7R)	Unknown	H7R	Unknown	No

ORF Number	Gene Family Name	Category	VACV-Cop ORF	Specific Function	Encapsidated
CPXV-BR-121	Large_capping_enzyme	RNA metabolism	D1R	capping of mRNA transcripts	Yes
CPXV-BR-124	Virion_core_(Cop_D3R)	Structure/Assembly	D3R	IV formation	Yes
CPXV-BR-126	NTPase_DNA_replication	DNA metabolism	D5R	Essential for DNA replication	No
CPXV-BR-127	Morph_VETF_s_early transcription_factor_small	RNA metabolism	D6R	Trascription factor	Yes
CPXV-BR-131	mutT_motif_NPH_PPH_RNA_levels_regulator	RNA metabolism	D10R	mRNA de-capping activity to regulate transription	No
CPXV-BR-132	NPH_I_Helicase_virion	RNA metabolism	D11L	Helicase, Transcription termination	Yes
CPXV-BR-142	Virion_Morphogenesis_(Cop_A6L)	Structure/Assembly	A6L	Involved in morphogenesis, found in core	Yes
CPXV-BR-144	VITF_3_34kda_subunit (Cop_A8R)	RNA metabolism	A8R	transcription factor subunit	No
CPXV-BR-146	P4a_precursor	Structure/Assembly	A10L	Major core protein	Yes
CPXV-BR-154	IMV_MP_PO4_(Cop_A17L)	Structure/Assembly	A17L	viral membrane morhpogenesis	Yes
CPXV-BR-155	DNA_Helicase_transcription	RNA metabolism	A18R	DNA helicase, elongation and termination of transcription	Yes
CPXV-BR-156	Unknown_(Cop_A19L)	DNA metabolism	A19L	Unknown	No
CPXV-BR-159	DNA_Processivity_factor	DNA metabolism	A20R	Processivity factor for DNA polymerase	No
CPXV-BR-160	Holliday_junction_resolvase	DNA metabolism	A22R	concatemer resolution, necessary for DNA replication	Yes
CPXV-BR-169	RNA_pol_35(RPO35)	RNA metabolism	A29L	Component of RNA polymerase	Yes
CPXV-BR-179	Unknown_(Cop_A37R)	Unknown	A37R	Unknown	No

ORF Number	Gene Family Name	Category	VACV-Cop ORF	Specific Function	Encapsidated
CPXV-BR-186	Membrane_glycoprotein_class_I	Unknown	A43R	Unknown, expressed late	No
CPXV-BR-188	Hydroxysteroid_dehydrogenase	Host Response Modifier	A44L	steroid synthesis, inhibition of inflammation	No
CPXV-BR-195	Unknown_(Cop_A51R)	Unknown	A51R	Unknown	No
CPXV-BR-196	Intracellular_TLR_and_IL_1 signaling_inhibitor_(Cop_A52R)	Host Response Modifier	A52R	Inhibits TLR signaling	No
CPXV-BR-205	Complement_control_CD46_EEV	Structure/Assembly	B5R	IEV formation, actin tail formation, not complement regulator, major target of neutralizing antibodies	Yes
CPXV-BR-208	IFN_gamma_receptor	Host Response Modifier	B19R	Binds type I interferons, required for virulence in ECTV, antigen	No
