# Supplementary material for: Genes in the terminal regions of orthopoxvirus genomes experience adaptive molecular evolution
Source: BMC Genomics. 2011 May 23;12:261. doi: 10.1186/1471-2164-12-261 (PMC3123329; doi:10.1186/1471-2164-12-261)
Supplement: Additional File 5 — Proportions and omega values of significant genes. [file 1471-2164-12-261-S5.PDF]

Proportions and omega values of significant genes

ORF Number	Gene Family	VACV-Cop ORF	Significant Model	p2 M2	w2 M2	p1 M8	w M8
CPXV-BR-021	EGF_Growth_factor	C11R	M2a & M8	0.081	4.307	0.134	2.832
CPXV-BR-023	Ubiquitin_Ligase_Host_defense_modulator	n/a	M8	0.072	1.000	0.019	2.798
CPXV-BR-024	IL_18_BP_(Bsh_D7L)	n/a	M2a & M8	0.073	3.859	0.082	3.581
CPXV-BR-025	Ankyrin_Host_Range_(Bang_D8L)	n/a	M8	0.204	1.752	0.203	1.756
CPXV-BR-027	Ankyrin_(Cop_C9L)	C9L	M8	0.297	1.497	0.295	1.503
CPXV-BR-035	Kelch_like_(Cop_C2L)	C2L	M2a & M8	0.038	5.610	0.040	5.472
CPXV-BR-036	Unknown_(Cop_C1L)	C1L	M2a & M8	0.023	12.309	0.023	12.308
CPXV-BR-044	Nicking_Joining_Enzyme_(Cop_K4L)	K4L	M8	0.028	1.000	0.007	6.515
CPXV-BR-046	Unknown_(Cop_K7R)	K7R	M2a & M8	0.009	10.875	0.009	10.892
CPXV-BR-048	Apoptosis_inhibitor_(mitochondrial_associated)	F1L	M2a & M8	0.140	3.163	0.139	3.173
CPXV-BR-050	Kelch_like_(Cop_F3L)	F3L	M8	0.057	1.000	0.036	1.939
CPXV-BR-053	36kDa_major_membrane_protein_(Cop_F5L)	F5L	M8	0.020	4.175	0.028	3.737
CPXV-BR-054	Unknown_(Cop_F6L)	F6L	M2a & M8	0.029	6.957	0.031	6.784
CPXV-BR-055	Unknown_(Cop_F7L)	F7L	M2a & M8	0.129	8.343	0.129	8.397
CPXV-BR-056	Cytoplasmic_protein_(Cop_F8L)	F8L	M2a & M8	0.017	12.533	0.049	4.045

ORF Number	Gene Family	VACV-Cop ORF	Significant Model	p2 M2	w2 M2	p1 M8	w M8
CPXV-BR-058	Ser_Thr_kinase_Morph_(Cop_F10L)	F10L	M8	0.047	1.000	0.019	1.000
CPXV-BR-061	IEV_associated_(Cop_F12L)	F12L	M8	0.072	1.000	0.043	1.162
CPXV-BR-063	Unknown_(Cop_F14L)	F14L	M2a & M8	0.037	10.015	0.037	8.433
CPXV-BR-066	Unknown_Conserved_(Cop_F15L)	F15L	M8	0.068	1.000	0.051	1.000
CPXV-BR-069	Poly_(A)_polymerase_large_(VP55)	E1L	M8	0.048	1.000	0.002	5.067
CPXV-BR-070	Unknown_(Cop_E2L)	E2L	M8	0.102	1.000	0.021	1.667
CPXV-BR-071	IFN_resistance_PKR_inhibitor_(Z_DNA_binding)	E3L	M8	0.150	1.000	0.019	4.874
CPXV-BR-072	RNA_pol_(RPO30)	E4L	M8	0.058	1.000	0.009	9.994
CPXV-BR-073	Virosome_component	E5R	M8	0.064	1.000	0.032	2.854
CPXV-BR-074	Unknown_(Cop_E6R)	E6R	M8	0.043	1.000	0.018	1.000
CPXV-BR-076	ER_localized_MP(Cop_E8R)	E8R	M8	0.014	1.000	0.004	5.395
CPXV-BR-079	Virion_core_protein_(Cop_E11L)	E11L	M8	0.056	1.000	0.014	4.644
CPXV-BR-080	Unknown_(Cop_O1L)	O1L	M8	0.071	1.000	0.006	4.706
CPXV-BR-083	DNA_binding_protein_(Cop_I1L)	I1L	M8	0.050	1.000	0.005	2.277
CPXV-BR-084	Unknown_(Cop_I2L)	I2L	M8	0.067	1.000	0.019	20.793
CPXV-BR-085	DNA_binding_phosphoprotein_(Cop_I3L)	I3L	M8	0.069	1.000	0.019	1.662
CPXV-BR-086	Ribonucleotide_Reductase_large_subunit	I4L	M8	0.034	1.000	0.071	1.298

ORF Number	Gene Family	VACV-Cop ORF	Significant Model	p2 M2	w2 M2	p1 M8	w M8
CPXV-BR-090	RNA_helicase_NPH_II	I8R	M8	0.050	1.000	0.023	1.997
CPXV-BR-095	Unknown_(Cop_G5R)	G5R	M8	0.070	1.000	0.015	3.570
CPXV-BR-098	Virion_assembly_protein_(Cop_G7L)	G7L	M8	0.069	1.000	0.037	1.000
CPXV-BR-103	Unknown_(Cop_L2R)	L2R	M8	0.032	2.851	0.065	2.381
CPXV-BR-104	Internal_Virion_Protein_(Cop_L3L)	L3L	M8	0.049	1.000	0.014	4.009
CPXV-BR-105	Core_package_transcription	L4R	M8	0.026	1.000	0.041	1.000
CPXV-BR-115	IMV_heparin_binding_surface_protein	H3L	M8	0.049	1.000	0.025	2.177
CPXV-BR-116	RAP94_(RNA_pol_assoc_protein)	H4L	M8	0.036	1.000	0.010	5.455
CPXV-BR-117	VLTF_4_(late_transcription_factor_4)	H5R	M8	0.106	1.000	0.110	1.294
CPXV-BR-120	Unknown_(Cop_H7R)	H7R	M8	0.057	1.000	0.029	1.000
CPXV-BR-121	Large_capping_enzyme	D1R	M8	0.035	1.000	0.004	3.535
CPXV-BR-124	Virion_core_(Cop_D3R)	D3R	M8	0.096	1.000	0.037	2.869
CPXV-BR-126	NTPase_DNA_replication	D5R	M8	0.024	1.000	0.010	1.000
CPXV-BR-127	Morph_VETF_s_early_transcription_factoe_small	D6R	M8	0.022	1.000	0.006	1.000
CPXV-BR-129	Carbonic_anhydrase_Virion	D8L	M2a & M8	0.022	6.144	0.052	3.854
CPXV-BR-131	mutT_motif_NPH_PPH_RNA_levels_regulator	D10R	M8	0.005	5.946	0.009	4.295
CPXV-BR-132	NPH_I_Helicase_virion	D11L	M8	0.020	1.000	0.008	1.174

ORF Number	Gene Family	VACV-Cop ORF	Significant Model	p2 M2	w2 M2	p1 M8	w M8
CPXV-BR-140	Core_protein_(Cop_A4L)	A4L	M2a & M8	0.026	4.472	0.087	2.440
CPXV-BR-142	Virion_Morphogenesis_(Cop_A6L)	A6L	M8	0.020	1.000	0.003	9.023
CPXV-BR-144	VITF_3_34kda_subunit_(Cop_A8R)	A8R	M8	0.023	1.000	0.028	1.000
CPXV-BR-145	Membrane_protein_(Cop_A9L)	A9L	M2a & M8	0.023	8.066	0.069	3.153
CPXV-BR-146	P4a_precursor	A10L	M8	0.093	1.000	0.023	1.969
CPXV-BR-154	IMV_MP_PO4_(Cop_A17L)	A17L	M8	0.046	1.000	0.052	3.588
CPXV-BR-155	DNA_Helicase_transcription	A18R	M8	0.056	1.000	0.016	1.800
CPXV-BR-156	Unknown_(Cop_A19L)	A19L	M8	0.035	1.000	0.013	6.906
CPXV-BR-159	DNA_Processivity_factor	A20R	M8	0.050	1.000	0.002	7.517
CPXV-BR-160	Holliday_junction_resolvase	A22R	M8	0.039	1.000	0.037	2.300
CPXV-BR-162	RNA_pol_132(RPO132)	A24R	M2a & M8	0.001	13.197	0.001	11.112
CPXV-BR-169	RNA_pol_35(RPO35)	A29L	M8	0.023	1.000	0.003	12.374
CPXV-BR-171	Unknown_(YMTV_120.5L)	A30.5L	M2a	0.046	5.769	0.044	6.570
CPXV-BR-172	Unknown_(Cop_A31R)	A31R	M2a & M8	0.034	6.633	0.082	2.794
CPXV-BR-179	Unknown_(Cop_A37R)	A37R	M8	0.052	1.000	0.009	5.032
CPXV-BR-182	Semaphorin	A39R	M2a & M8	0.027	10.417	0.027	10.159
CPXV-BR-186	Membrane_glycoprotein_class_I	A43R	M8	0.200	1.844	0.198	1.854

ORF Number	Gene Family	VACV-Cop ORF	Significant Model	p2 M2	w2 M2	p1 M8	w M8
CPXV-BR-188	Hydroxysteroid_dehydrogenase	A44L	M8	0.080	1.000	0.017	2.477
CPXV-BR-191	Unknown_(Cop_A47L)	A47L	M2a & M8	0.146	2.769	0.180	2.543
CPXV-BR-192	Thymidylate_kinase	A48R	M2a & M8	0.015	7.440	0.015	7.406
CPXV-BR-195	Unknown_(Cop_A51R)	A51R	M8	0.064	1.000	0.007	3.498
CPXV-BR-196	Intracellular_TLR_and_IL_1_signaling_inhibitor_(	A52R	M8	0.091	1.000	0.069	1.884
CPXV-BR-200	Hemagglutinin	A56R	M2a & M8	0.033	3.298	0.110	2.079
CPXV-BR-203	Schlafen_(Cop_B2R)	B2R	M2a & M8	0.055	3.066	0.065	2.884
CPXV-BR-204	Ankyrin_(Cop_B4R)	B4R	M2a & M8	0.017	6.947	0.017	6.905
CPXV-BR-205	Complement_control_CD46_EEV	B5R	M8	0.178	1.000	0.128	1.091
CPXV-BR-208	IFN_gamma_receptor	B19R	M8	0.061	1.000	0.036	1.519
CPXV-BR-212	Ser_Thr_Kinase_(Cop_B12R)	B12R	M2a & M8	0.007	14.958	0.008	13.444
CPXV-BR-215	IL_1_beta_receptor	B15R	M2a & M8	0.035	4.384	0.047	3.788
CPXV-BR-216	Unknown_(Cop_B17L)	B17R	M2a & M8	0.039	3.451	0.053	3.011
