# Supplementary material for: Genes in the terminal regions of orthopoxvirus genomes experience adaptive molecular evolution
Source: BMC Genomics. 2011 May 23;12:261. doi: 10.1186/1471-2164-12-261 (PMC3123329; doi:10.1186/1471-2164-12-261)
Supplement: Additional File 6 — Sites in significant genes (models M2a and M8) under diversifying selection determined by Bayes empirical Bayes analysis. [file 1471-2164-12-261-S6.PDF]

Sites in significant genes (models M2a and M8) under diversifying selection determined by Bayes Empirical Bayes analysis

Gene	Model M2a Site	Model M2a Pr(w>1)[†]	Model M2a Post Mean +/- SE[††]	Model M8 Site	Model M8 Pr(w>1)[†]	Model M8 Post Mean +/- SE[††]
Ankyrin_(Cop_B4R)						
				I 13	0.727	2.596 +- 1.374
				R 22	0.759	2.717 +- 1.413
	L 23	0.888	4.438 +- 1.965	L 23	0.959*	3.333 +- 1.07
				V 39	0.579	2.123 +- 1.402
	T 80	0.859	4.321 +- 2.026	T 80	0.95	3.306 +- 1.093
				H 101	0.637	2.319 +- 1.447
	G 104	0.87	4.365 +- 2.005	G 104	0.953*	3.316 +- 1.085
				R 127	0.726	2.61 +- 1.432
				C 154	0.77	2.733 +- 1.348
	V 225	0.842	4.246 +- 2.059	V 225	0.944	3.29 +- 1.107
				S 262	0.698	2.525 +- 1.452
	K 275	0.993**	4.79 +- 1.646	K 275	0.998**	3.439 +- 0.951
				L 298	0.642	2.334 +- 1.445
	A 301	0.5	2.801 +- 2.14	A 301	0.761	2.722 +- 1.411
				D 351	0.694	2.49 +- 1.379
	R 362	0.903	4.42 +- 1.888	R 362	0.981*	3.39 +- 1.003
	P 404	0.897	4.474 +- 1.943	P 404	0.962*	3.341 +- 1.062
				K 451	0.744	2.65 +- 1.367
				T 476	0.722	2.596 +- 1.434
				T 495	0.712	2.546 +- 1.375
				A 527	0.705	2.539 +- 1.44
Apoptosis_inhibitor_(mitochondrial_associated)						
	Y 11	0.969*	6.458 +- 1.989	Y 11	0.987*	6.395 +- 1.666
	V 22	0.715	4.951 +- 2.938	V 22	0.779	5.19 +- 2.77
	P 40	0.937	6.301 +- 2.198	P 40	0.961*	6.262 +- 1.881
	P 48	0.996**	6.622 +- 1.797	P 48	0.998**	6.458 +- 1.564
	H 60	0.606	4.258 +- 3	H 60	0.677	4.556 +- 2.972
	Q 96	0.575	4.061 +- 2.993	Q 96	0.644	4.352 +- 3.007

Gene	Model M2a Site	Model M2a Pr(w>1)[†]	Model M2a Post Mean +/- SE[††]	Model M8 Site	Model M8 Pr(w>1)[†]	Model M8 Post Mean +/- SE[††]
	M 124	0.833	5.541 +- 2.55	M 124	0.931	6.035 +- 2.055
	Y 141	0.523	3.653 +- 2.802	Y 141	0.641	4.254 +- 2.896
	A 173	0.62	4.317 +- 2.964	A 173	0.7	4.677 +- 2.917
	P 216	0.93	6.257 +- 2.23	P 216	0.961*	6.256 +- 1.882
	P 217	0.933	6.28 +- 2.214	P 217	0.962*	6.264 +- 1.874
				I 221	0.527	3.623 +- 3.013
	T 224	0.786	5.25 +- 2.675	T 224	0.898	5.835 +- 2.237
Carbonic_anhydrase_Virion						
	A 19	0.742	4.695 +- 2.717	A 19	0.914	4.009 +- 1.522
	S 124	0.815	5.102 +- 2.601	S 124	0.935	4.089 +- 1.458
				S 143	0.739	3.367 +- 1.904
				Q 230	0.792	3.523 +- 1.754
	A 246	0.982*	5.879 +- 2.006	A 246	0.995**	4.283 +- 1.228
	E 248	0.513	3.392 +- 2.657	E 248	0.835	3.697 +- 1.694
				A 261	0.693	3.12 +- 1.832
				G 272	0.674	3.105 +- 1.953
				L 293	0.779	3.483 +- 1.784
Core Protein (Cop A4L)						
	A 63	0.827	3.604 +- 1.662	A 63	0.915	3.145 +- 1.049
				S 65	0.626	2.337 +- 1.49
				Q 81	0.54	2.077 +- 1.498
	P 88	0.523	2.555 +- 1.825	P 88	0.653	2.414 +- 1.475
	A 136	0.752	3.285 +- 1.679	A 136	0.902	3.099 +- 1.067
				P 142	0.555	2.12 +- 1.499
	K 147	0.55	2.611 +- 1.715	K 147	0.737	2.642 +- 1.355
	S 192	0.7	3.09 +- 1.682	S 192	0.879	3.032 +- 1.117
	A 234	0.969*	4.009 +- 1.349	A 234	0.992**	3.332 +- 0.792
	S 243	0.853	3.689 +- 1.622	S 243	0.929	3.18 +- 1.012
Cytoplasmic_protein_(Cop_F8L)						

Gene	Model M2a Site	Pr(w>1)†	Post Mean +/- SE††	Model M8 Site	Pr(w>1)†	Post Mean +/- SE††
	R 11	0.645	5.787 +- 3.873	R 11	0.803	6.077 +- 3.36
	H 20	0.994**	8.296 +- 2.04	H 20	0.998**	7.394 +- 2.242
EGF_Growth_factor						
	S 29	0.982*	5.307 +- 1.911	S 29	0.991**	4.417 +- 1.482
	D 57	0.657	3.798 +- 2.498	D 57	0.788	3.646 +- 1.966
	F 72	0.6	3.246 +- 2.174	F 72	0.842	3.725 +- 1.746
	K 93	0.68	3.809 +- 2.393	K 93	0.844	3.829 +- 1.84
	V 107	0.698	4.009 +- 2.5	V 107	0.817	3.77 +- 1.931
	T 116	0.943	5.129 +- 2.049	T 116	0.975*	4.358 +- 1.539
				P 139	0.591	2.83 +- 2.056
Hemagglutinin						
				T 2	0.658	2.011 +- 1.083
				R 3	0.571	1.848 +- 1.171
	E 93	0.925	3.006 +- 1.201	E 93	0.981*	2.742 +- 0.764
				Y 149	0.62	1.947 +- 1.153
				S 153	0.554	1.787 +- 1.144
	T 193	0.557	2.175 +- 1.427	T 193	0.666	2.072 +- 1.199
	C 284	0.507	1.99 +- 1.233	C 284	0.679	2.087 +- 1.143
IL-1 Beta Receptor						
	V 6	0.859	3.911 +- 1.562	V 6	0.957*	3.774 +- 0.958
	Q 19	0.935	4.238 +- 1.402	Q 19	0.970*	3.821 +- 0.908
	A 23	0.93	4.217 +- 1.416	A 23	0.969*	3.816 +- 0.913
	P 24	0.983*	4.394 +- 1.219	P 24	0.995**	3.892 +- 0.776
	S 82	0.665	3.19 +- 1.776	S 82	0.836	3.378 +- 1.342
	L 126	0.609	2.948 +- 1.745	L 126	0.831	3.352 +- 1.333
	G 201	0.988*	4.409 +- 1.199	G 201	0.996**	3.895 +- 0.77
				D 228	0.588	2.535 +- 1.594
	V 230	0.732	3.414 +- 1.709	V 230	0.901	3.586 +- 1.161
	A 241	0.867	3.954 +- 1.551	A 241	0.954*	3.764 +- 0.976

Gene	Model M2a			Model M8		
	Site	Pr(w>1)†	Post Mean +/- SE††	Site	Pr(w>1)†	Post Mean +/- SE††
	T 326	0.677	3.22 +- 1.758	T 326	0.852	3.428 +- 1.3
IL_18_BP_(Bsh_D7L)						
	K 2	0.717	2.849 +- 1.715	K 2	0.94	2.627 +- 0.995
	P 16	0.793	3.282 +- 1.95	P 16	0.941	2.66 +- 1.061
				N 29	0.625	1.804 +- 1.044
				R 38	0.558	1.644 +- 1.024
				F 45	0.768	2.142 +- 0.977
	N 48	0.56	2.252 +- 1.447	N 48	0.887	2.457 +- 0.958
				S 61	0.52	1.545 +- 1.169
				A 65	0.657	1.883 +- 1.051
				I 84	0.595	1.731 +- 1.037
				R 87	0.656	1.878 +- 1.049
	V 89	0.857	3.491 +- 1.908	V 89	0.970*	2.729 +- 1.033
	Q 92	0.847	3.463 +- 1.921	Q 92	0.963*	2.714 +- 1.042
				A 113	0.847	2.335 +- 0.933
	L 115	0.831	3.387 +- 1.91	L 115	0.963*	2.711 +- 1.034
				I 118	0.673	1.912 +- 0.982
	Y 119	0.803	3.376 +- 2.014	Y 119	0.938	2.658 +- 1.079
				V 123	0.668	1.901 +- 0.982
	C 124	0.946	3.874 +- 1.908	C 124	0.986*	2.778 +- 1.033
Kelch_like_(Cop_C2L)						
	I 5	0.9	5.632 +- 3.087	I 5	0.934	4.981 +- 2.598
	V 78	0.922	5.744 +- 2.101	V 78	0.958*	5.089 +- 1.618
	S 99	0.848	5.372 +- 1.969	S 99	0.88	4.744 +- 1.47
	R 111	0.992**	6.122 +- 2.351	R 111	0.997**	5.262 +- 1.881
	N 154	0.987*	6.098 +- 1.527	N 154	0.993**	5.248 +- 1.204
	K 283	0.724	4.622 +- 1.568	K 283	0.829	4.462 +- 1.231
	D 306	0.914	5.69 +- 2.569	D 306	0.964*	5.11 +- 2.011
	V 407	0.986*	6.089 +- 1.993	V 407	0.995**	5.254 +- 1.43
				T 475	0.518	2.914 +- 1.216
	R 476	0.939	5.836 +- 1.57	R 476	0.956*	5.078 +- 2.316

Gene	Model M2a Site	Pr(w>1)[†]	Post Mean +/- SE[††]	Model M8 Site	Pr(w>1)[†]	Post Mean +/- SE[††]
RNA pol 132 (RPO132)						
	A 1164	0.984*	6.663 +- 3.499			
Schlafen_(Cop_B2R)						
				F 9	0.581	1.315 +- 0.913
	S 37	0.765	2.139 +- 1.027	S 37	0.951*	2.043 +- 0.629
				N 41	0.769	1.697 +- 0.762
	D 44	0.679	1.935 +- 0.955	D 44	0.926	1.995 +- 0.641
	D 48	0.501	1.566 +- 0.791	D 48	0.795	1.747 +- 0.749
				Y 54	0.574	1.301 +- 0.913
	Y 56	0.781	2.178 +- 1.037	Y 56	0.955*	2.051 +- 0.627
				L 66	0.638	1.43 +- 0.91
	L 84	0.575	1.722 +- 0.889	L 84	0.849	1.85 +- 0.72
	V 92	0.585	1.744 +- 0.901	V 92	0.855	1.862 +- 0.716
	G 118	0.524	1.614 +- 0.822	G 118	0.815	1.785 +- 0.739
	T 123	0.788	2.172 +- 1.012	T 123	0.965*	2.067 +- 0.611
	A 126	0.789	2.174 +- 1.009	A 126	0.965*	2.068 +- 0.61
				N 149	0.825	1.804 +- 0.695
	S 150	0.841	2.301 +- 1.034	S 150	0.975*	2.086 +- 0.606
				S 152	0.618	1.414 +- 0.794
				V 167	0.527	1.206 +- 0.908
				R 182	0.707	1.581 +- 0.783
	P 192	0.809	2.244 +- 1.049	P 192	0.961*	2.064 +- 0.623
				L 201	0.776	1.712 +- 0.759
				E 204	0.642	1.439 +- 0.911
				V 208	0.689	1.547 +- 0.79
	S 213	0.657	1.91 +- 0.986	S 213	0.892	1.934 +- 0.693
				K 230	0.515	1.182 +- 0.907
	S 244	0.502	1.569 +- 0.794	S 244	0.796	1.749 +- 0.749
	R 246	0.575	1.723 +- 0.889	R 246	0.85	1.852 +- 0.719
				F 256	0.761	1.682 +- 0.765
	D 257	0.627	1.799 +- 0.867	D 257	0.923	1.983 +- 0.625

Gene	Model M2a Site	Model M2a Pr(w>1)[†]	Model M2a Post Mean +/- SE[††]	Model M8 Site	Model M8 Pr(w>1)[†]	Model M8 Post Mean +/- SE[††]
				R 264	0.605	1.364 +- 0.913
	V 267	0.599	1.77 +- 0.906	V 267	0.869	1.888 +- 0.702
	M 269	0.83	2.268 +- 1.022	M 269	0.974*	2.084 +- 0.605
	S 271	0.595	1.767 +- 0.915	S 271	0.861	1.873 +- 0.713
				S 272	0.787	1.732 +- 0.753
				S 275	0.619	1.414 +- 0.797
				M 277	0.659	1.489 +- 0.792
				L 278	0.583	1.32 +- 0.914
				K 288	0.642	1.436 +- 0.887
				G 291	0.776	1.71 +- 0.757
	Y 298	0.828	2.29 +- 1.054	Y 298	0.965*	2.072 +- 0.62
	E 300	0.517	1.591 +- 0.777	E 300	0.835	1.822 +- 0.705
				E 303	0.719	1.603 +- 0.781
	R 314	0.826	2.285 +- 1.055	R 314	0.965*	2.071 +- 0.62
	L 320	0.543	1.655 +- 0.849	L 320	0.828	1.81 +- 0.732
	P 321	0.862	2.366 +- 1.054	P 321	0.972*	2.084 +- 0.617
				E 322	0.689	1.547 +- 0.79
				E 330	0.715	1.597 +- 0.782
	A 332	0.633	1.83 +- 0.904	A 332	0.91	1.963 +- 0.649
				G 333	0.723	1.612 +- 0.779
				S 334	0.536	1.225 +- 0.91
	L 338	0.615	1.813 +- 0.94	L 338	0.871	1.894 +- 0.706
				A 339	0.793	1.743 +- 0.75
	E 352	0.55	1.658 +- 0.812	E 352	0.869	1.885 +- 0.673
	P 353	0.777	2.169 +- 1.035	P 353	0.954*	2.049 +- 0.627
				N 355	0.669	1.51 +- 0.792
				H 364	0.785	1.728 +- 0.756
				Q 378	0.597	1.347 +- 0.915
	D 391	0.505	1.578 +- 0.805	D 391	0.795	1.748 +- 0.752
				V 399	0.632	1.416 +- 0.891
	Q 413	0.673	1.943 +- 0.994	Q 413	0.901	1.951 +- 0.684
				Y 415	0.602	1.358 +- 0.913
				L 416	0.618	1.39 +- 0.912

Gene	Model M2a			Model M8		
	Site	Pr(w>1)[†]	Post Mean +/- SE[††]	Site	Pr(w>1)[†]	Post Mean +/- SE[††]
				K 417	0.733	1.631 +- 0.777
	S 421	0.539	1.638 +- 0.803	S 421	0.86	1.868 +- 0.68
				D 422	0.676	1.523 +- 0.793
	Y 426	0.922	2.478 +- 1.022	Y 426	0.990*	2.113 +- 0.595
	K 428	0.629	1.826 +- 0.908	K 428	0.907	1.958 +- 0.653
	R 431	0.687	1.955 +- 0.964	R 431	0.929	2 +- 0.64
				E 456	0.715	1.596 +- 0.784
				V 463	0.513	1.178 +- 0.906
				A 477	0.766	1.691 +- 0.763
				S 488	0.64	1.455 +- 0.795
	M 491	0.538	1.643 +- 0.842	M 491	0.824	1.802 +- 0.734
				D 497	0.577	1.336 +- 0.795
	H 502	0.933	2.503 +- 1.017	H 502	0.991**	2.116 +- 0.594
Semaphorin						
	K 72	0.969*	6.833 +- 2.057	K 72	0.989*	5.288 +- 1.523
	Y 115	0.976*	6.875 +- 2.003	Y 115	0.991**	5.297 +- 1.511
	G 117	0.879	6.257 +- 2.576	G 117	0.968*	5.189 +- 1.635
	Y 145	0.844	6.087 +- 2.738	Y 145	0.942	5.081 +- 1.771
				G 179	0.639	3.595 +- 2.468
				S 262	0.577	3.278 +- 2.471
				A 365	0.697	3.907 +- 2.445
				R 370	0.591	3.346 +- 2.47
				R 394	0.58	3.29 +- 2.466
	Y 398	0.971*	6.835 +- 2.038	Y 398	0.993**	5.303 +- 1.497
	L 399	0.901	6.397 +- 2.479	L 399	0.960*	5.154 +- 1.693
Ser Thr Kinase (Cop B12R)						
	L 104	0.959*	5.094 +- 2.767	L 104	0.967*	2.988 +- 1.702
	R 133	0.844	4.697 +- 2.944	R 133	0.851	2.789 +- 1.808
	D 170	0.798	4.443 +- 2.96	D 170	0.829	2.729 +- 1.809
				R 200	0.645	2.073 +- 1.477

Gene	Model M2a Site	Pr(w>1)[†]	Post Mean +/- SE[††]	Model M8 Site	Pr(w>1)[†]	Post Mean +/- SE[††]
Thymidylate_kinase						
	P 52	0.963*	6.426 +- 2.253	P 52	0.986*	5.9 +- 2.129
	A 126	0.874	5.917 +- 2.674	A 126	0.925	5.572 +- 2.436
	A 171	0.999**	6.615 +- 2.036	A 171	1.000**	5.965 +- 2.055
Unknown (Cop A31R)						
	A 2	0.998**	6.395 +- 1.823	A 2	0.999**	5.187 +- 1.528
	S 36	0.782	5.165 +- 2.708	S 36	0.853	4.478 +- 2.134
	A 91	0.985*	6.327 +- 1.909	A 91	0.995**	5.168 +- 1.554
	N 126	0.838	5.459 +- 2.543	N 126	0.903	4.701 +- 1.948
	N 129	0.937	6.047 +- 2.174	N 129	0.972*	5.055 +- 1.671
Unknown_(Cop_A47L)						
	S 18	0.738	2.962 +- 1.763	S 18	0.885	2.824 +- 1.256
				W 30	0.612	1.982 +- 1.405
	R 40	0.985*	3.824 +- 1.638	R 40	0.997**	3.162 +- 1.106
				Q 44	0.535	1.744 +- 1.357
	V 54	0.525	2.138 +- 1.387	V 54	0.751	2.36 +- 1.228
	E 68	0.819	3.219 +- 1.72	E 68	0.945	3.003 +- 1.172
	A 71	0.769	3.003 +- 1.661	A 71	0.927	2.938 +- 1.175
				I 88	0.552	1.784 +- 1.172
				T 93	0.646	2.078 +- 1.393
	S 110	0.621	2.491 +- 1.576	S 110	0.82	2.594 +- 1.252
	N 114	0.542	2.198 +- 1.42	N 114	0.765	2.405 +- 1.232
	V 122	0.581	2.336 +- 1.496	V 122	0.793	2.5 +- 1.242
				A 139	0.7	2.201 +- 1.214
				L 146	0.566	1.837 +- 1.378
	Y 163	0.82	3.255 +- 1.766	Y 163	0.934	2.978 +- 1.202
	S 164	0.64	2.562 +- 1.609	S 164	0.832	2.635 +- 1.254
	A 172	0.602	2.418 +- 1.541	A 172	0.807	2.55 +- 1.249
	N 173	0.617	2.476 +- 1.569	N 173	0.817	2.585 +- 1.251
	G 196	0.605	2.443 +- 1.573	G 196	0.807	2.555 +- 1.259
	M 236	0.598	2.344 +- 1.398	M 236	0.842	2.623 +- 1.15

Gene	Model M2a Site	Pr(w>1)[†]	Post Mean +/- SE[††]	Model M8 Site	Pr(w>1)[†]	Post Mean +/- SE[††]
	R 237	0.523	2.13 +- 1.428	R 237	0.737	2.321 +- 1.264
	H 240	0.857	3.379 +- 1.746	H 240	0.957*	3.045 +- 1.166
Unknown_(Cop_B17L)						
	V 53	0.651	2.372 +- 1.32	V 53	0.91	2.759 +- 0.888
	R 173	0.512	2.009 +- 1.218	R 173	0.799	2.486 +- 1.072
	P 178	0.657	2.492 +- 1.485	P 178	0.831	2.578 +- 1.079
	L 183	0.634	2.362 +- 1.362	L 183	0.874	2.677 +- 0.97
	S 184	0.854	3.025 +- 1.422	S 184	0.958*	2.88 +- 0.79
	L 205	0.883	3.078 +- 1.383	L 205	0.975*	2.916 +- 0.737
	A 223	0.892	3.103 +- 1.375	A 223	0.977*	2.921 +- 0.73
	I 250	0.564	2.123 +- 1.236	I 250	0.869	2.656 +- 0.956
				Y 252	0.552	1.858 +- 1.181
				D 253	0.7	2.238 +- 1.219
				N 259	0.732	2.315 +- 1.131
	T 266	0.89	3.071 +- 1.352	T 266	0.982*	2.929 +- 0.714
				M 273	0.544	1.831 +- 1.275
				E 302	0.583	1.931 +- 1.267
Unknown_(Cop_C1L)						
	Q 8	0.522	4.995 +- 4.332	Q 8	0.625	5.555 +- 4.133
	Y 19	1.000**	8.958 +- 1.468	Y 19	1.000**	8.531 +- 1.616
	D 25	0.992**	8.903 +- 1.608	D 25	0.998**	8.515 +- 1.654
	R 58	0.836	7.613 +- 3.231	R 58	0.93	7.972 +- 2.52
				E 100	0.636	5.498 +- 3.855
	A 119	0.999**	8.955 +- 1.474	A 119	1.000**	8.531 +- 1.617
Unknown_(Cop_C8L)						
	A 3	0.82	2.495 +- 1.029	A 3	0.929	2.66 +- 0.848
	S 14	0.638	2.085 +- 1.056	S 14	0.74	2.196 +- 1.138
	V 15	0.825	2.502 +- 1.021	V 15	0.933	2.667 +- 0.839
	F 16	0.732	2.285 +- 1.019	F 16	0.877	2.532 +- 0.936
	S 21	0.946	2.753 +- 0.931	S 21	0.990*	2.79 +- 0.699

Gene	Model M2a Site	Pr(w>1)†	Post Mean +/- SE††	Model M8 Site	Pr(w>1)†	Post Mean +/- SE††
				K 70	0.657	1.985 +- 1.061
	Q 99	0.662	2.124 +- 1	Q 99	0.821	2.393 +- 1.004
	S 102	0.832	2.48 +- 0.968	S 102	0.958*	2.718 +- 0.768
	P 129	0.787	2.416 +- 1.031	P 129	0.91	2.615 +- 0.884
	E 135	0.796	2.412 +- 0.994	E 135	0.934	2.664 +- 0.824
				D 155	0.565	1.747 +- 1.189
Unknown_(Cop_F14L)						
	G 11	0.989*	7.363 +- 2.061	G 11	0.990**	5.766 +- 2.043
	T 25	0.629	4.781 +- 3.278	T 25	0.673	3.818 +- 2.546
	M 26	0.993**	7.387 +- 2.021	M 26	0.993**	5.772 +- 2.033
Unknown (Cop F6L)						
	F 68	0.511	3.671 +- 3.148	F 68	0.592	4.007 +- 3.196
	T 69	0.995**	6.888 +- 2.306	T 69	0.998**	6.687 +- 2.297
Unknown_(Cop_F7L)						
	S 7	0.516	3.951 +- 3.399	S 7	0.665	4.199 +- 3.097
				R 11	0.516	3.255 +- 2.935
				C 13	0.52	3.246 +- 2.902
	N 20	0.719	5.23 +- 3.309	N 20	0.844	5.25 +- 2.853
	V 25	0.633	4.616 +- 3.338	V 25	0.79	4.874 +- 2.918
	A 27	0.716	5.211 +- 3.311	A 27	0.843	5.24 +- 2.854
	Y 32	0.756	5.493 +- 3.258	Y 32	0.867	5.401 +- 2.808
	Y 33	0.849	6.154 +- 3.032	Y 33	0.919	5.75 +- 2.672
				I 38	0.585	3.673 +- 3.023
	F 44	0.568	4.315 +- 3.457	F 44	0.706	4.469 +- 3.101
	E 45	0.988*	7.002 +- 2.352	E 45	0.995**	6.171 +- 2.362
				R 47	0.58	3.639 +- 3.015
				T 75	0.606	3.808 +- 3.05
Unknown_(Cop_K7R)						
	V 63	0.941	5.685 +- 2.885	V 63	0.949	4.726 +- 2.718

Gene	Model M2a			Model M8		
	**Site**	**Pr(w>1)** †	**Post Mean +/- SE** ††	**Site**	**Pr(w>1)** †	**Post Mean +/- SE** ††
Unknown (YMTV_120.5L)						
	V 7	0.902	5.888 +- 2.889	V 7	0.925	6.229 +- 2.823

† posterior probability of w>1
†† predicted w +/- standard error
* p<0.05
** p<0.01
