# Supplementary material for: Genes in the terminal regions of orthopoxvirus genomes experience adaptive molecular evolution
Source: BMC Genomics. 2011 May 23;12:261. doi: 10.1186/1471-2164-12-261 (PMC3123329; doi:10.1186/1471-2164-12-261)
Supplement: Additional File 7 — Sites in significant genes (model M8 only) under diversifying selection determined by Bayes Empirical Bayes analysis. [file 1471-2164-12-261-S7.PDF]

Sites in significant genes (model M8 only) under diversifying selection determined by Bayes Empirical Bayes analysis

	Model M8		
Gene†	Site	Pr(w>1)††	Post Mean +/- SE†††
36kDa_major_membrane_protein_(Cop_F5L)			
	V 5	0.973*	2.876 +- 0.999
	H 34	0.56	1.892 +- 1.335
	D 36	0.951*	2.829 +- 1.037
	H 42	0.804	2.505 +- 1.237
	L 44	0.572	1.891 +- 1.254
	A 229	0.523	1.791 +- 1.314
	E 236	0.839	2.571 +- 1.181
	E 238	0.771	2.421 +- 1.258
	A 249	0.705	2.248 +- 1.282
	V 254	0.531	1.78 +- 1.226
	L 259	0.573	1.927 +- 1.34
Ankyrin_(Cop_C9L)			
	N 3	0.926	1.536 +- 0.373
	K 5	0.673	1.232 +- 0.521
	C 19	0.77	1.353 +- 0.483
	G 28	0.55	1.081 +- 0.545
	N 36	0.516	0.997 +- 0.627
	I 58	0.626	1.175 +- 0.536
	L 59	0.804	1.395 +- 0.469
	V 64	0.827	1.423 +- 0.457
	V 78	0.748	1.325 +- 0.494
	C 79	0.842	1.439 +- 0.419
	E 86	0.844	1.444 +- 0.421
	S 93	0.814	1.407 +- 0.465
	L 96	0.741	1.316 +- 0.497
	I 101	0.833	1.43 +- 0.454
	V 119	0.651	1.205 +- 0.528
	Q 137	0.899	1.506 +- 0.396
	Y 154	0.584	1.089 +- 0.619
	L 160	0.622	1.143 +- 0.61
	D 161	0.608	1.123 +- 0.615
	D 162	0.913	1.522 +- 0.388
	Y 163	0.873	1.478 +- 0.432
	Q 189	0.519	1 +- 0.627
	G 191	0.804	1.395 +- 0.441
	Y 192	0.723	1.294 +- 0.504
	A 193	0.598	1.11 +- 0.616
	H 205	0.557	1.053 +- 0.623
	E 206	0.637	1.188 +- 0.536
	R 210	0.815	1.409 +- 0.468
	E 216	0.783	1.368 +- 0.473
	H 217	0.66	1.217 +- 0.522

	Model M8		
Gene[†]	Site	Pr(w>1)[††]	Post Mean +/- SE[†††]
	L 224	0.746	1.323 +- 0.492
	A 236	0.536	1.024 +- 0.628
	F 238	0.567	1.066 +- 0.623
	S 243	0.848	1.448 +- 0.417
	G 244	0.62	1.167 +- 0.533
	H 245	0.963*	1.574 +- 0.347
	L 247	0.565	1.064 +- 0.629
	S 252	0.703	1.269 +- 0.506
	Q 258	0.598	1.109 +- 0.616
	F 259	0.689	1.252 +- 0.515
	N 261	0.694	1.259 +- 0.519
	Y 264	0.832	1.429 +- 0.455
	V 271	0.644	1.196 +- 0.53
	K 273	0.788	1.374 +- 0.477
	S 276	0.788	1.375 +- 0.477
	E 277	0.889	1.496 +- 0.405
	H 278	0.883	1.489 +- 0.404
	S 289	0.64	1.191 +- 0.525
	L 290	0.816	1.409 +- 0.464
	I 294	0.501	0.973 +- 0.642
	I 296	0.784	1.371 +- 0.485
	S 297	0.875	1.481 +- 0.437
	T 298	0.77	1.354 +- 0.492
	D 305	0.627	1.176 +- 0.529
	S 312	0.937	1.549 +- 0.374
	E 313	0.589	1.129 +- 0.536
	H 318	0.678	1.238 +- 0.512
	Y 321	0.793	1.38 +- 0.474
	D 330	0.784	1.374 +- 0.454
	T 334	0.829	1.425 +- 0.424
	Q 346	0.921	1.53 +- 0.379
	K 347	0.594	1.134 +- 0.545
	D 349	0.825	1.42 +- 0.458
	R 351	0.729	1.301 +- 0.497
	N 364	0.691	1.254 +- 0.515
	V 365	0.805	1.395 +- 0.469
	N 368	0.642	1.193 +- 0.535
	I 373	0.7	1.266 +- 0.516
	L 383	0.648	1.178 +- 0.604
	S 384	0.598	1.11 +- 0.616
	I 385	0.548	1.04 +- 0.625
	R 386	0.639	1.166 +- 0.606
	S 388	0.665	1.201 +- 0.598
	D 402	0.722	1.3 +- 0.471
	C 412	0.743	1.319 +- 0.493
	H 417	0.654	1.209 +- 0.523
	E 420	0.897	1.504 +- 0.395

Gene[†]	Model M8 Site	Pr(w>1)[††]	Post Mean +/- SE[†††]
	S 421	0.61	1.125 +- 0.624
	V 424	0.81	1.401 +- 0.466
	S 425	0.942	1.553 +- 0.37
	T 440	0.721	1.291 +- 0.503
	S 445	0.732	1.305 +- 0.5
	G 449	0.716	1.286 +- 0.506
	M 454	0.522	1.002 +- 0.633
	H 456	0.656	1.211 +- 0.524
	A 457	0.881	1.486 +- 0.403
	K 468	0.511	0.989 +- 0.628
	I 472	0.728	1.3 +- 0.498
	K 485	0.664	1.222 +- 0.522
	S 491	0.875	1.48 +- 0.432
	N 492	0.57	1.07 +- 0.622
	D 493	0.651	1.181 +- 0.603
	H 495	0.669	1.207 +- 0.597
	L 497	0.648	1.178 +- 0.604
	G 499	0.711	1.279 +- 0.503
	N 500	0.779	1.363 +- 0.481
	K 501	0.57	1.07 +- 0.621
	T 502	0.821	1.415 +- 0.46
	L 506	0.684	1.23 +- 0.575
	K 507	0.818	1.413 +- 0.435
	D 523	0.751	1.329 +- 0.495
	T 524	0.803	1.393 +- 0.467
	A 540	0.801	1.39 +- 0.469
	R 543	0.752	1.329 +- 0.488
	M 550	0.912	1.522 +- 0.388
	I 555	0.632	1.157 +- 0.603
	K 558	0.872	1.476 +- 0.408
	H 562	0.753	1.331 +- 0.488
	R 569	0.941	1.551 +- 0.361
	H 570	0.73	1.302 +- 0.499
	V 572	0.934	1.545 +- 0.368
	E 575	0.87	1.473 +- 0.408
	A 577	0.825	1.421 +- 0.426
	K 578	0.824	1.421 +- 0.435
	R 579	0.934	1.545 +- 0.373
	E 597	0.767	1.349 +- 0.484
	N 599	0.731	1.305 +- 0.5
	N 603	0.914	1.524 +- 0.386
	E 606	0.871	1.474 +- 0.407
	A 607	0.813	1.406 +- 0.468
	A 610	0.963*	1.573 +- 0.352
	N 612	0.724	1.297 +- 0.509
	I 617	0.66	1.216 +- 0.524
	I 622	0.719	1.29 +- 0.515

Gene[†]	Model M8 Site	Pr(w>1)[††]	Post Mean +/- SE[†††]
	A 630	0.586	1.093 +- 0.619
Ankyrin_Host_Range_(Bang_D8L)			
	D 3	0.76	1.499 +- 0.685
	A 11	0.742	1.465 +- 0.674
	R 20	0.711	1.408 +- 0.661
	E 49	0.504	1.04 +- 0.749
	H 63	0.572	1.163 +- 0.775
	Q 68	0.808	1.595 +- 0.711
	L 69	0.795	1.568 +- 0.704
	T 70	0.54	1.105 +- 0.764
	P 71	0.773	1.537 +- 0.773
	L 72	0.584	1.186 +- 0.779
	G 73	0.531	1.089 +- 0.761
	V 90	0.764	1.508 +- 0.686
	Y 97	0.774	1.527 +- 0.692
	D 102	0.521	1.071 +- 0.756
	T 107	0.833	1.645 +- 0.726
	D 117	0.533	1.093 +- 0.761
	K 120	0.531	1.089 +- 0.76
	D 128	0.513	1.058 +- 0.755
	F 129	0.62	1.254 +- 0.792
	K 135	0.911	1.789 +- 0.719
	H 137	0.808	1.595 +- 0.712
	I 165	0.519	1.068 +- 0.757
	D 171	0.811	1.602 +- 0.716
	A 176	0.864	1.71 +- 0.741
	Q 183	0.666	1.343 +- 0.809
	R 190	0.618	1.251 +- 0.792
	L 198	0.651	1.314 +- 0.805
	S 202	0.532	1.09 +- 0.76
	S 208	0.654	1.32 +- 0.805
	S 210	0.627	1.268 +- 0.795
	S 212	0.558	1.139 +- 0.77
	C 213	0.5	1.034 +- 0.748
	N 215	0.937	1.841 +- 0.728
	N 228	0.537	1.101 +- 0.762
	S 231	0.532	1.091 +- 0.76
	S 267	0.584	1.187 +- 0.779
	G 278	0.907	1.779 +- 0.715
	H 309	0.83	1.639 +- 0.724
	G 310	0.903	1.773 +- 0.714
	S 327	0.785	1.547 +- 0.697
	V 337	0.578	1.176 +- 0.777
	S 349	0.835	1.65 +- 0.727
	S 354	0.796	1.57 +- 0.704
	Y 358	0.634	1.282 +- 0.798

Gene[†]	Model M8		
	Site	Pr(w>1)[††]	Post Mean +/- SE[†††]
	E 361	0.763	1.506 +- 0.687
	A 364	0.748	1.477 +- 0.677
	G 373	0.777	1.532 +- 0.693
	- 393	0.704	1.419 +- 0.822
	G 394	0.518	1.065 +- 0.755
	A 396	0.622	1.257 +- 0.793
	V 397	0.603	1.222 +- 0.786
	R 417	0.93	1.826 +- 0.726
	T 424	0.915	1.797 +- 0.72
	Q 427	0.556	1.134 +- 0.769
	F 429	0.522	1.072 +- 0.756
	R 430	0.523	1.074 +- 0.756
	D 431	0.552	1.128 +- 0.77
	K 465	0.574	1.168 +- 0.776
	E 480	0.507	1.046 +- 0.752
	R 504	0.809	1.597 +- 0.712
	N 510	0.506	1.044 +- 0.752
	C 521	0.567	1.155 +- 0.773
	R 527	0.521	1.071 +- 0.756
	I 529	0.521	1.072 +- 0.758
	A 536	0.948	1.861 +- 0.729
	Y 537	0.538	1.101 +- 0.763
	R 544	0.507	1.046 +- 0.752
	A 546	0.638	1.289 +- 0.799
	D 551	0.585	1.189 +- 0.78
	I 553	0.548	1.121 +- 0.766
	S 554	0.669	1.35 +- 0.81
	K 563	0.587	1.192 +- 0.783
	H 564	0.88	1.743 +- 0.746
	I 571	0.595	1.207 +- 0.784
	E 575	0.528	1.084 +- 0.76
	E 578	0.586	1.191 +- 0.781
	I 583	0.504	1.041 +- 0.75
	M 589	0.516	1.063 +- 0.755
	D 596	0.779	1.54 +- 0.7
	A 606	0.636	1.285 +- 0.798
	L 611	0.621	1.256 +- 0.793
	A 613	0.853	1.686 +- 0.736
	S 617	0.632	1.276 +- 0.797
	V 626	0.567	1.155 +- 0.773
	N 632	0.927	1.821 +- 0.726
	N 638	0.768	1.516 +- 0.69
	K 639	0.81	1.599 +- 0.713
	N 643	0.859	1.699 +- 0.739
	T 659	0.864	1.71 +- 0.741
	Y 662	0.638	1.29 +- 0.799

	Model M8		
**Gene**[†]	**Site**	**Pr(w>1)**[††]	**Post Mean +/- SE**[†††]
Complement_control_CD46_EEV			
	S 50	0.879	1.658 +- 0.666
Core_package_transcription			
	I 181	0.918	4.679 +- 2.815
DNA_binding_protein_(Cop_I1L)			
	A 114	0.977*	2.788 +- 1.759
DNA Helicase Transcription			
	I 101	0.503	1.028 +- 0.517
	L 103	0.824	1.375 +- 0.345
	A 241	0.906	1.447 +- 0.27
	A 264	0.818	1.358 +- 0.379
	D 311	0.521	1.024 +- 0.545
	P 348	0.515	1.068 +- 0.48
	E 460	0.725	1.268 +- 0.44
	S 472	0.655	1.171 +- 0.521
	V 473	0.88	1.42 +- 0.312
	T 475	0.721	1.263 +- 0.442
DNA_Processivity_factor			
	V 53	0.53	1.241 +- 0.808
	A 416	0.904	1.873 +- 1.161
ER_localized_MP(Cop_E8R)			
	A 4	0.988*	5.742 +- 2.663
Holliday_junction_resolvase			
	M 12	0.974*	3.054 +- 1.397
	S 47	0.799	2.464 +- 1.378
	A 106	0.885	2.858 +- 1.548
Hydroxysteroid_dehydrogenase			
	T 190	0.715	1.336 +- 0.57
	E 239	0.935	1.557 +- 0.417
	Y 250	0.632	1.25 +- 0.565
	D 330	0.757	1.389 +- 0.527
IEV_associated_(Cop_F12L)			
	I 5	0.976*	1.869 +- 0.654
	T 7	0.964*	1.859 +- 0.667
	R 58	0.614	1.353 +- 0.666
	R 121	0.843	1.726 +- 0.774
	A 210	0.834	1.715 +- 0.78

Gene[†]	Model M8 Site	Pr(w>1)[††]	Post Mean +/- SE[†††]
	A 225	0.615	1.401 +- 0.884
	S 342	0.522	1.241 +- 0.857
	Q 463	0.622	1.36 +- 0.673
	D 544	0.791	1.657 +- 0.8
	A 559	0.572	1.329 +- 0.876
IFN gamma receptor			
	E 112	0.733	1.351 +- 0.509
	T 160	0.7	1.324 +- 0.49
IFN Resistance PKR Inhibitor (Z-DNA binding)			
	D 11	0.759	3.543 +- 1.979
	V 30	0.617	2.901 +- 2.027
	A 79	1.000**	4.618 +- 1.381
	S 83	1.000**	4.619 +- 1.381
IMV_MP_PO4_(Cop_A17L)			
	M 41	0.959*	4.204 +- 1.333
	K 165	0.869	3.862 +- 1.666
	R 166	0.873	3.88 +- 1.654
	T 170	0.511	2.381 +- 2.201
	N 182	0.791	3.537 +- 1.832
	V 187	0.878	3.898 +- 1.641
	D 188	0.758	3.396 +- 1.879
Internal_Virion_Protein_(Cop_L3L)			
	R 22	0.999**	3.937 +- 1.535
	Q 34	0.669	2.66 +- 1.928
	P 48	0.872	3.52 +- 1.832
	R 55	0.606	2.382 +- 1.876
Intracellular_TLR_and_IL_1_signaling_inhibitor_(Cop_A52R)			
	S 10	0.794	2.165 +- 1.206
	S 57	0.635	1.797 +- 1.181
	G 71	0.747	2.024 +- 1.117
	K 150	0.747	2.054 +- 1.222
	D 169	0.654	1.828 +- 1.219
Kelch-like (Cop F3L)			
	P 61	0.562	1.097 +- 0.562
	A 149	0.823	1.386 +- 0.413
	K 195	0.901	1.461 +- 0.332
	R 245	0.548	1.08 +- 0.565
	V 315	0.649	1.208 +- 0.506
	Y 423	0.609	1.15 +- 0.551
	D 478	0.597	1.165 +- 0.491

Gene[†]	Model M8 Site	Pr(w>1)[††]	Post Mean +/- SE[†††]
Large_capping_enzyme			
	G 28	0.946	1.77 +- 0.887
	K 202	0.976*	1.803 +- 0.806
	S 325	0.932	1.743 +- 0.932
Membrane_glycoprotein_class_I			
	M 4	0.61	1.495 +- 1.321
	S 14	0.538	1.365 +- 0.846
	A 20	0.76	1.836 +- 0.888
	E 31	0.585	1.461 +- 0.982
	V 33	0.713	1.742 +- 0.915
	H 39	0.828	1.976 +- 0.998
	I 45	0.528	1.346 +- 0.976
	N 54	0.649	1.588 +- 0.932
	L 68	0.584	1.471 +- 0.916
	T 69	0.848	2.075 +- 0.95
	A 123	0.765	1.829 +- 1.079
	K 135	0.524	1.331 +- 0.944
	K 143	0.921	2.208 +- 0.859
	I 146	0.904	2.169 +- 1.034
	Y 160	0.646	1.584 +- 1.029
	K 162	0.756	1.832 +- 0.948
	D 165	0.919	2.208 +- 0.987
	S 183	0.852	2.068 +- 1.04
Morph_VETF_s_early_transcription_factor_small			
	S 562	0.751	3.47 +- 2.788
mutT_motif_NPH_PPH_RNA_levels_regulator			
	E 26	0.605	2.379 +- 1.815
	Y 110	0.58	2.166 +- 1.624
	T 114	0.999**	3.97 +- 1.676
Nicking_Joining_Enzyme_(Cop_K4L)			
	P 3	0.907	1.508 +- 0.651
	R 93	0.529	1.044 +- 0.546
	M 193	0.511	1.005 +- 0.558
	S 265	0.970*	1.569 +- 0.616
	C 409	0.957*	1.558 +- 0.626
NTPase DNA Replication			
	T 4	0.827	1.455 +- 0.58
	D 45	0.886	1.535 +- 0.561
	G 106	0.543	1.085 +- 0.575
P4a Precursor			
	Q 39	0.853	1.433 +- 0.417

Gene[†]	Model M8 Site	Pr(w>1)[††]	Post Mean +/- SE[†††]
	S 83	0.891	1.469 +- 0.407
	A 217	0.937	1.52 +- 0.334
	D 225	0.787	1.365 +- 0.477
	N 268	0.771	1.336 +- 0.501
	D 274	0.929	1.511 +- 0.349
	I 283	0.749	1.295 +- 0.56
	F 284	0.739	1.282 +- 0.567
	D 285	0.79	1.356 +- 0.488
	C 292	0.978*	1.556 +- 0.292
	E 306	0.915	1.496 +- 0.353
	Q 312	0.741	1.285 +- 0.565
	I 331	0.64	1.155 +- 0.612
	A 334	0.901	1.479 +- 0.397
Poly_(A)_polymerase_large_(VP55)			
	L 10	0.992**	3.983 +- 2.331
RAP94_(RNA_pol_assoc_protein)			
	A 17	0.656	1.257 +- 0.574
	E 313	0.579	1.166 +- 0.564
	I 620	0.743	1.384 +- 0.641
	C 623	0.774	1.441 +- 0.708
	I 624	0.962*	1.719 +- 0.738
	Q 644	0.971*	1.728 +- 0.739
Ribonucleotide_Reductase_large_subunit			
	Y 9	0.581	1.277 +- 0.851
	A 60	0.633	1.347 +- 0.844
	E 99	0.727	1.521 +- 0.764
	V 123	0.821	1.663 +- 0.677
	E 184	0.852	1.703 +- 0.694
	S 185	0.598	1.313 +- 0.809
	S 211	0.627	1.36 +- 0.755
	I 224	0.842	1.693 +- 0.675
	T 268	0.952*	1.847 +- 0.556
	S 306	0.687	1.447 +- 0.804
	D 361	0.75	1.545 +- 0.747
	Q 373	0.589	1.297 +- 0.822
	K 378	0.619	1.302 +- 0.917
	S 382	0.927	1.816 +- 0.592
	G 458	0.768	1.587 +- 0.748
	R 459	0.846	1.706 +- 0.678
	L 463	0.631	1.368 +- 0.816
	L 521	0.886	1.764 +- 0.637
	T 556	0.73	1.525 +- 0.758
	S 582	0.818	1.664 +- 0.69

Gene[†]	Model M8<br>Site	Pr(w>1)[††]	Post Mean +/- SE[†††]
	R 588	0.619	1.339 +- 0.811
	E 652	0.845	1.696 +- 0.684
	A 660	0.692	1.458 +- 0.803
	A 744	0.661	1.402 +- 0.811
	P 758	0.978*	1.877 +- 0.524
RNA_helicase_NPH_II			
	T 33	0.913	1.526 +- 0.407
	V 82	0.625	1.182 +- 0.603
	T 87	0.699	1.288 +- 0.519
	T 88	0.946	1.559 +- 0.38
	R 142	0.573	1.154 +- 0.57
	S 173	0.625	1.212 +- 0.519
	R 460	0.515	1.069 +- 0.599
	Q 482	0.908	1.519 +- 0.411
	I 592	0.675	1.26 +- 0.492
	E 595	0.814	1.421 +- 0.482
	K 611	0.980*	1.588 +- 0.346
	N 625	0.511	1.081 +- 0.492
	E 631	0.725	1.311 +- 0.53
RNA_pol_(RPO30)			
	A 21	0.996**	5.082 +- 2.736
	S 38	0.681	3.864 +- 3.318
	A 248	0.792	4.046 +- 2.966
Ubiquitin Ligase Host defense modulator			
	M 71	0.627	1.287 +- 0.615
	N 95	0.792	1.482 +- 0.581
	N 137	0.794	1.489 +- 0.595
Unknown_(Cop_A19L)			
	V 6	0.995**	6.948 +- 2.388
Unknown_(Cop_A37R)			
	D 92	0.811	1.388 +- 0.456
	L 240	0.575	1.159 +- 0.477
	N 263	0.736	1.322 +- 0.461
Unknown_(Cop_A51R)			
	D 45	0.735	1.292 +- 0.458
	I 71	0.572	1.11 +- 0.539
	A 107	0.623	1.178 +- 0.502
	H 140	0.960*	1.504 +- 0.242
	H 231	0.554	1.085 +- 0.552
	E 287	0.646	1.202 +- 0.495
	S 306	0.589	1.125 +- 0.545

Gene†	Model M8 Site	Pr(w>1)††	Post Mean +/- SE†††
Unknown_(Cop_E2L)			
	K 30	0.565	1.128 +- 0.61
	S 88	0.659	1.26 +- 0.541
	R 147	0.604	1.203 +- 0.461
	P 350	0.659	1.264 +- 0.52
	N 491	0.913	1.523 +- 0.416
	R 656	0.863	1.476 +- 0.452
	D 659	0.851	1.464 +- 0.459
	A 696	0.663	1.25 +- 0.598
	D 700	0.812	1.425 +- 0.498
Unknown_(Cop_E6R)			
	A 102	0.522	1.034 +- 0.608
	E 127	0.633	1.18 +- 0.561
	A 194	0.759	1.334 +- 0.545
	P 251	0.912	1.499 +- 0.431
	T 428	0.609	1.16 +- 0.586
	S 446	0.749	1.32 +- 0.491
Unknown_(Cop_G5R)			
	V 309	0.663	2.773 +- 2.043
	T 254	0.868	3.199 +- 1.443
	V 309	0.976*	3.475 +- 1.124
	D 330	0.894	3.21 +- 1.303
	S 411	0.755	2.806 +- 1.619
Unknown (Cop H7R)			
	N 20	0.572	1.307 +- 0.797
	S 72	0.801	1.655 +- 0.815
Unknown (Cop I2L)			
	V 37	0.998**	8.289 +- 2.03
Unknown (Cop O1L)			
	R 478	0.638	1.408 +- 0.666
	L 101	0.784	1.372 +- 0.472
	E 239	0.807	1.4 +- 0.456
	D 282	0.601	1.17 +- 0.545
	Y 313	0.763	1.356 +- 0.47
	R 478	0.986*	1.558 +- 0.298
Unknown_(Cop_L2R)			
	E 13	0.506	1.652 +- 1.141
	V 72	0.641	2.597 +- 2.219
Unknown_Conserved_(Cop_F15L)			
	S 58	0.817	1.545 +- 0.707

Gene[†]	Model M8 Site	Pr(w>1)[††]	Post Mean +/- SE[†††]
	N 62	0.539	1.16 +- 0.697
	E 112	0.635	1.276 +- 0.83
Virion_assembly_protein_(Cop_G7L)			
	D 12	0.585	1.12 +- 0.477
	S 151	0.561	1.08 +- 0.503
	V 247	0.714	1.234 +- 0.459
Virion_core_(Cop_D3R)			
	S 34	0.889	2.154 +- 0.957
	E 97	0.685	1.785 +- 1.06
	I 103	0.718	1.857 +- 1.065
	D 159	0.587	1.572 +- 1.022
Virion_core_protein_(Cop_E11L)			
	A 18	0.806	3.721 +- 2.434
	E 29	0.991**	4.396 +- 2.151
Virion_Morphogenesis_(Cop_A6L)			
	V 95	1.000**	7.436 +- 2.236
Virosome_component			
	E 27	0.704	1.581 +- 0.761
	I 33	0.531	1.312 +- 0.738
	R 76	0.82	1.73 +- 0.69
	P 85	0.667	1.526 +- 0.77
	F 93	0.586	1.403 +- 0.772
	Q 108	0.763	1.662 +- 0.738
	R 134	0.874	1.797 +- 0.654
	R 175	0.535	1.324 +- 0.764
	R 199	0.611	1.448 +- 0.75
	E 209	0.515	1.294 +- 0.761
	S 227	0.703	1.579 +- 0.778
VITF_3_34kda_subunit_(Cop_A8R)			
	Q 145	0.718	1.518 +- 0.908
	H 169	0.527	1.229 +- 0.997
	E 247	0.908	1.895 +- 0.979
VLTF_4_(late_transcription_factor_4)			
	A 55	0.639	1.376 +- 0.586
	V 135	0.56	1.29 +- 0.591

† No significant sites were predicted in CPXV DNA_binding_phosphoprotein_ (Cop_I3L), NPH I Helicase Virion and Ser/Thr Kinase Morph (Cop F10L). Significant sitesthat aligned with gaps in CPXV-BR were predicted in homologs.

Gene†	Model M8		
	Site	Pr(w>1)††	Post Mean +/- SE†††

†† posterior probability of w>1

††† predicted w +/- standard error

* p<0.05

** p<0.01
